# Low-density neutrophil heterogeneity and spleen tyrosine kinase as therapeutic targets in sepsis

**DOI:** 10.1172/jci.insight.201057

**Published:** 2026-04-21

**Authors:** Heather L. Teague, Lauren Knabe, Raquel S. Da Cruz, Xianglan Yao, Kiana C. Allen, Trenton Williams, Cumhur Y. Demirkale, Merte Woldehanna, Ernest Evans, Amir Hobson, Jared D. Wilkinson, Steven D. Nathan, Christopher S. King, Jeffrey R. Strich

**Affiliations:** 1 Critical Care Medicine and Pulmonary Branch, National Heart Lung and Blood Institute, NIH, Bethesda, Maryland, USA.; 2 Critical Care Medicine Department, Clinical Center, NIH, Bethesda, Maryland, USA.; 3 Advanced Lung Disease and Lung Transplant Program, Inova Fairfax Hospital, Falls Church, Virginia, USA.

**Keywords:** Immunology, Inflammation, Neutrophils

## Abstract

Sepsis is a leading cause of death for which host-directed therapies are urgently needed. We performed high-dimensional flow cytometry, measurement of soluble biomarkers, and lipopolysaccharide (LPS) stimulation of neutrophils to characterize neutrophil heterogeneity and function in patients with sepsis. We observed that in patients with sepsis, low-density neutrophils (LDNs) are elevated and phenotypically diverse populations of innate immune cells with varying degrees of maturity and myeloperoxidase expression. Spleen tyrosine kinase (SYK) expression was found to be higher in whole blood neutrophils and LDNs of patients with sepsis compared with healthy donors. Importantly, SYK^+^ LDNs associated with increased levels of intracellular myeloperoxidase (MPO) and soluble biomarkers. Furthermore, SYK^+^ LDNs correlated with clinical outcomes of sepsis disease severity, including sequential organ failure assessment score, mechanical ventilation, and vasopressors. Functionally, the SYK inhibitor R406 suppressed changes in neutrophil features of activation from normal-density neutrophils and LDNs, including the SYK^+^ and SYK^–^ neutrophil subsets, and MPO release from LDNs following LPS stimulation of sepsis neutrophils. Combined, these results establish LDNs as a heterogenous population of neutrophils that express high levels of SYK and support SYK inhibition as a potentially novel therapeutic target aimed at suppressing overactive neutrophils in sepsis.

## Abstract 2

Low-density neutrophils and spleen tyrosine kinase are elevated and associate with disease severity in sepsis, providing a novel therapeutic target.

## Introduction

Sepsis is a global health priority that leads to an estimated 11 million deaths globally and 370,000 deaths in the United States per year (1, 2). In addition to high mortality, sepsis survivorship is associated with many long-term sequelae (3). Sepsis is defined as the dysregulated immune response that occurs in the setting of infection (4). Despite decades of research, there have not been substantive advances in the development of host-directed therapies targeting this dysregulated innate immune response (5). Neutrophils, which are activated by pathogen-associated molecular patterns and disease-associated molecular patterns, are an essential part of the innate immune response to clear invading pathogens (6). However, when neutrophils become overactive, many of their important end-effector functions, such as excessive degranulation, release of reactive oxygen species (ROS), expulsion of neutrophil extracellular traps (NETs), and adherence to endothelial cells, contribute to the dysregulated innate immune response and associated end-organ dysfunction, making them a plausible therapeutic target (7, 8).

It is becoming increasingly recognized that during critical illness neutrophils consist of heterogenous populations of cells with different phenotypic characteristics (9). One such subset of neutrophils are low-density neutrophils (LDNs). LDNs are a population of neutrophils that based on their physical properties separate into the peripheral blood mononuclear (PBMC) layer following density gradient centrifugation (10). LDNs are primarily described in chronic disease where they are thought to be hyperinflammatory (11). During sepsis, LDNs are described as dysfunctional, which is attributable to their impaired phagocytic activity, reduced migration, and prolonged lifespan (12). In totality, LDNs in sepsis are understudied, with a substantial portion of the literature focused on a subset of LDNs termed myeloid-derived suppressor cells that inhibit the adaptive immune responses during excessive inflammation (12–14). Nevertheless, LDNs themselves are not a homogeneous population of innate immune cells and therefore warrant more detailed characterization to determine if they are a potential therapeutic target during sepsis.

Spleen tyrosine kinase (SYK) is an intracellular non-receptor tyrosine kinase found in myeloid cells including neutrophils. SYK has been evaluated as a therapeutic target in critically ill patients with coronavirus disease 2019 (COVID-19) and mechanistically was found to impact neutrophil activation, including decreasing the proportion of circulating LDNs and shifting neutrophils to a more mature and less activated phenotype (15–20). Additionally, we have identified that LDNs and SYK expression are increased in a nonhuman primate model of bacterial sepsis and the proportion of SYK-expressing whole blood neutrophils (WBNs) and LDNs correlate with the degree of end-organ dysfunction (21).

We therefore sought to characterize WBNs and LDNs and associated SYK expression in human patients with sepsis. We observed that LDNs are increased during sepsis and represent a heterogenous population of innate immune cells that associate with disease severity. Furthermore, SYK expression is increased in both WBNs and LDNs, associates with soluble markers of disease severity, and associates with clinical end-organ dysfunction. Taken together, these data, along with our mechanistic work demonstrating that SYK inhibition can ameliorate activated sepsis neutrophils, suggest that SYK inhibition may be a potential therapeutic target in humans with sepsis (22).

## Results

### Patient characteristics.

**Figure 1:**
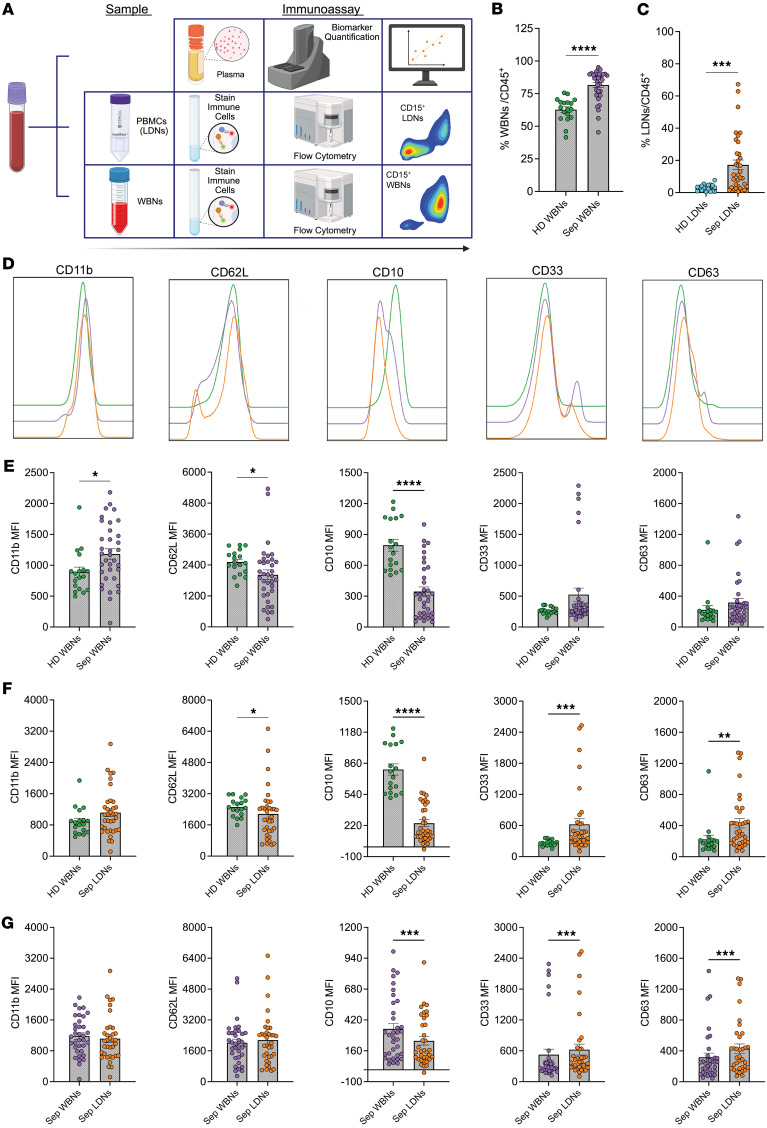
Characterization of WBNs and LDNs in patients with sepsis. (**A**) Schematic representation of methods and datasets to analyze WBNs and LDNs in healthy donors and patients with sepsis. (**B**) Bar graphs comparing the proportion of WBNs and (**C**) LDNs in healthy donors and patients with sepsis. (**D**) Histogram demonstrating the relative expression, MFI, of features between healthy donor WBNs (green) and sepsis WBNs (purple) and LDNs (orange). (**E**) Bar graphs comparing the relative expression of features between healthy donors and sepsis WBNs. (**F**) Bar graphs comparing the relative expression of features between healthy donor WBNs and sepsis LDNs. (**G**) Bar graphs comparing the relative expression of features between sepsis WBNs and LDNs. Data are represented as means ± SEM. Significance was determined using a Mann-Whitney test, a paired *t* test, or a Wilcoxon test and set at *P* < 0.05*, *P* < 0.01**, *P* < 0.001***, *P* < 0.0001****. PBMCs, peripheral blood mononuclear cells; WBNs, whole blood neutrophils; HD, healthy donor; Sep, sepsis; MFI, mean fluorescence intensity; LDNs, low-density neutrophils; SEM, standard error of the mean.

We performed flow cytometry on matched whole blood and PBMC fractions to capture LDNs from 18 healthy donors (HDs) and 35 patients with sepsis (Figure 1A). The mean age, sex, and race distributions were similar between HDs and patients with sepsis (Table 1). Among the patients with sepsis, intra-abdominal (29%) and pneumonia (23%) were the most common sites of infection. There was an equal distribution of polymicrobial (17%), Gram-negative (17%) and Gram-positive (17%) infections, while 8.6% were viral, 2.9% were fungal, and 37% were culture negative. A majority of the patients had septic shock (57%). At the time of blood draw, 71% were in the intensive care unit, 49% were on vasopressors, 23% were on mechanical ventilation, 60% had an acute kidney injury, and the mean SOFA score was 4.4. In-hospital mortality was 9% and the average length of stay was 9.6 days.

### Patients with sepsis have increased WBNs and LDNs compared with HDs.

High-dimensional flow cytometry analysis of whole blood demonstrated patients with sepsis had a higher proportion of CD15^+^ WBNs compared with HDs (*P* < 0.0001) (Figure 1B and Supplemental Figure 1A; supplemental material available online with this article; https://doi.org/10.1172/jci.insight.201057DS1). Likewise, patients with sepsis had a higher proportion of LDNs (17.2% vs. 3.0%) (*P* < 0.001) (Figure 1C and Supplemental Figure 1B). Comparing WBNs from patients with sepsis with HDs using traditional gating strategies identified that patients had lower CD10 (*P* < 0.0001; a marker of immature neutrophils), increased CD11b (*P* < 0.05), and lower expression of CD62L (*P* < 0.05) consistent with an activated phenotype, while there were no statistical differences in other features tested (Figure 1, D and E, and Supplemental Figure 2, A and B). Similarly, comparing HD WBNs with sepsis LDNs, sepsis LDNs had lower CD10 (*P* < 0.0001) and higher CD33 (*P* < 0.001) (markers consistent with immature neutrophils) and higher CD63 (*P* < 0.01) and lower CD62L (*P* < 0.05) (markers of degranulation and activation) (Figure 1, D and F, and Supplemental Figure 2, A and C). Despite the low frequency of LDNs in HDs, we next compared HD LDNs with sepsis LDNs and found that sepsis LDNs have lower CD10 (*P* < 0.0001) and higher CD33 (*P* < 0.05) (Supplemental Figure 3). Last, sepsis LDNs had lower CD10 (*P* < 0.001) and higher CD33 (*P* < 0.001), CD63 (*P* < 0.001), and CD15 (*P* < 0.0001) compared with sepsis WBNs (Figure 1, D and G, and Supplemental Figure 2, A and D).

**Figure 2:**
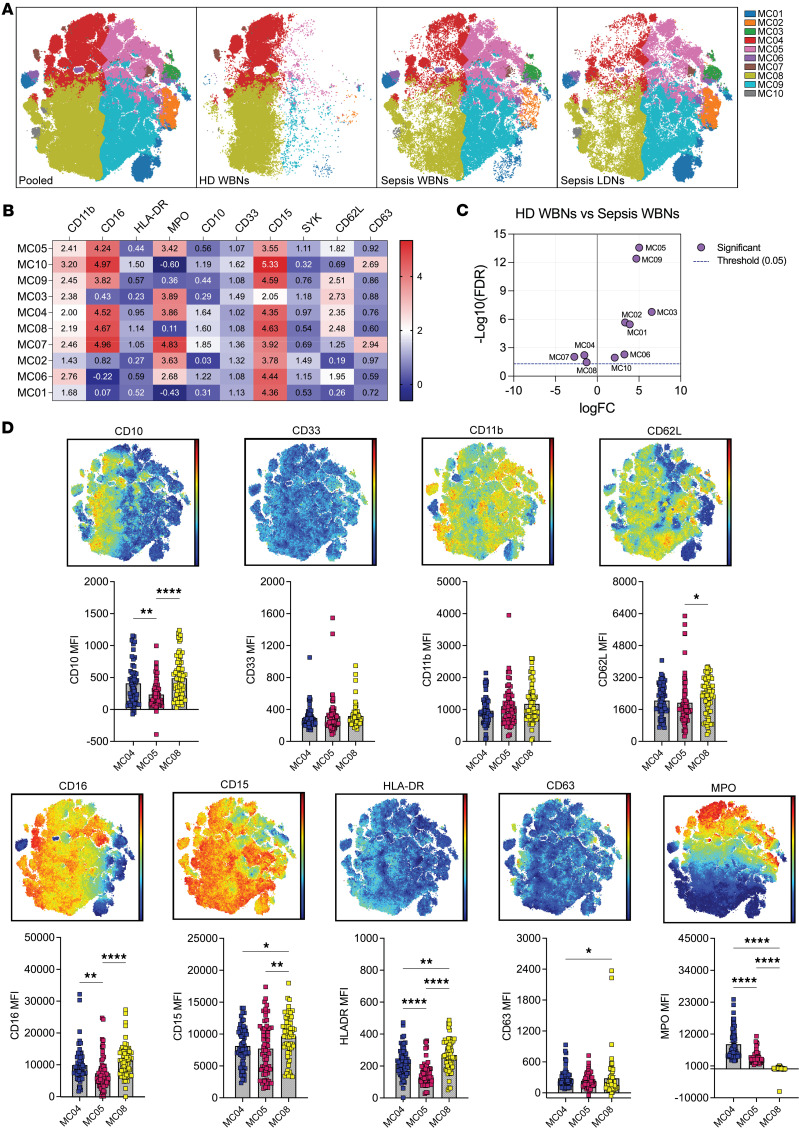
Characterization of sepsis and healthy donor neutrophils. (**A**) optSNE plot of flow cytometry data overlaid with FlowSOM MCs from all pooled samples, healthy donor WBNs, and sepsis WBNs and LDNs. (**B**) Heatmap representing the relative expression of each extracellular and intracellular feature between MCs. (**C**) Volcano plot comparing the logFC versus log10(FDR) between healthy donor WBNs and sepsis WBNs. Purple dots represent MCs that are statistically different between healthy donor and sepsis WBNs. (**D**) optSNE plots and bar graphs comparing the relative expression of each feature for MC04, MC05, and MC08. Data are represented as means ± SEM. Significance was determined using a 1-way ANOVA with a Tukey’s multiple comparisons test or a Kruskal-Wallis with a Dunn’s multiple-comparison test and set at *P* < 0.05*, *P* < 0.01**, *P* < 0.0001****. optSNE, optimized t-distributed stochastic neighbor embedding; FlowSOM, flow cytometry-specific self-organizing map; MC, metacluster; logFC, log fold-change; FDR, false discovery rate; WBNs, whole blood neutrophils; HD, healthy donor; LDNs, low-density neutrophils; SEM, standard error of the mean.

We next sought to determine differences in these neutrophil subsets at the population level and therefore performed dimensional reduction with clustering by flow cytometry-specific self-organizing map (FlowSOM) of CD15^+^ WBNs from 18 HDs and WBNs and LDNs from 23 patients with sepsis, which identified 10 metaclusters (MC) (Figure 2, A and B, and Supplemental Table 1). MC08 and MC04 were the predominant populations in the HDs, representing 49.9% and 46.0% of the HD WBNs, respectively (Supplemental Figure 4 and Supplemental Table 2). MC05 (logFC 5.03, FDR < 0.01), MC09 (logFC 4.69, FDR < 0.01), MC01 (logFC 3.89, FDR < 0.01), MC03 (logFC 6.52, FDR < 0.01), and MC02 (logFC 3.84, FDR < 0.01) were all significantly elevated in sepsis WBNs compared with HD WBNs (Figure 2C, Supplemental Figure 4, and Supplemental Table 3). MC05 demonstrated decreased CD10 (*P* < 0.01), CD16 (*P* < 0.01), MPO (*P* < 0.001), and HLA-DR (*P* < 0.0001) compared with MC04, along with increased MPO (*P* < 0.0001) and decreased CD10 (*P* < 0.0001), CD16 (*P* < 0.0001), CD62L (*P* < 0.05), CD15 (*P* < 0.01), and HLA-DR (*P* < 0.001) compared with MC08 (Figure 2, B and D). Similarly, MC09 expressed decreased CD10 (*P* < 0.001), CD16 (*P* < 0.001), and HLA-DR (*P* < 0.0001) compared with MC04 and MC08 and decreased MPO (*P* < 0.0001) compared with MC04 (Supplemental Figure 5).

**Figure 3:**
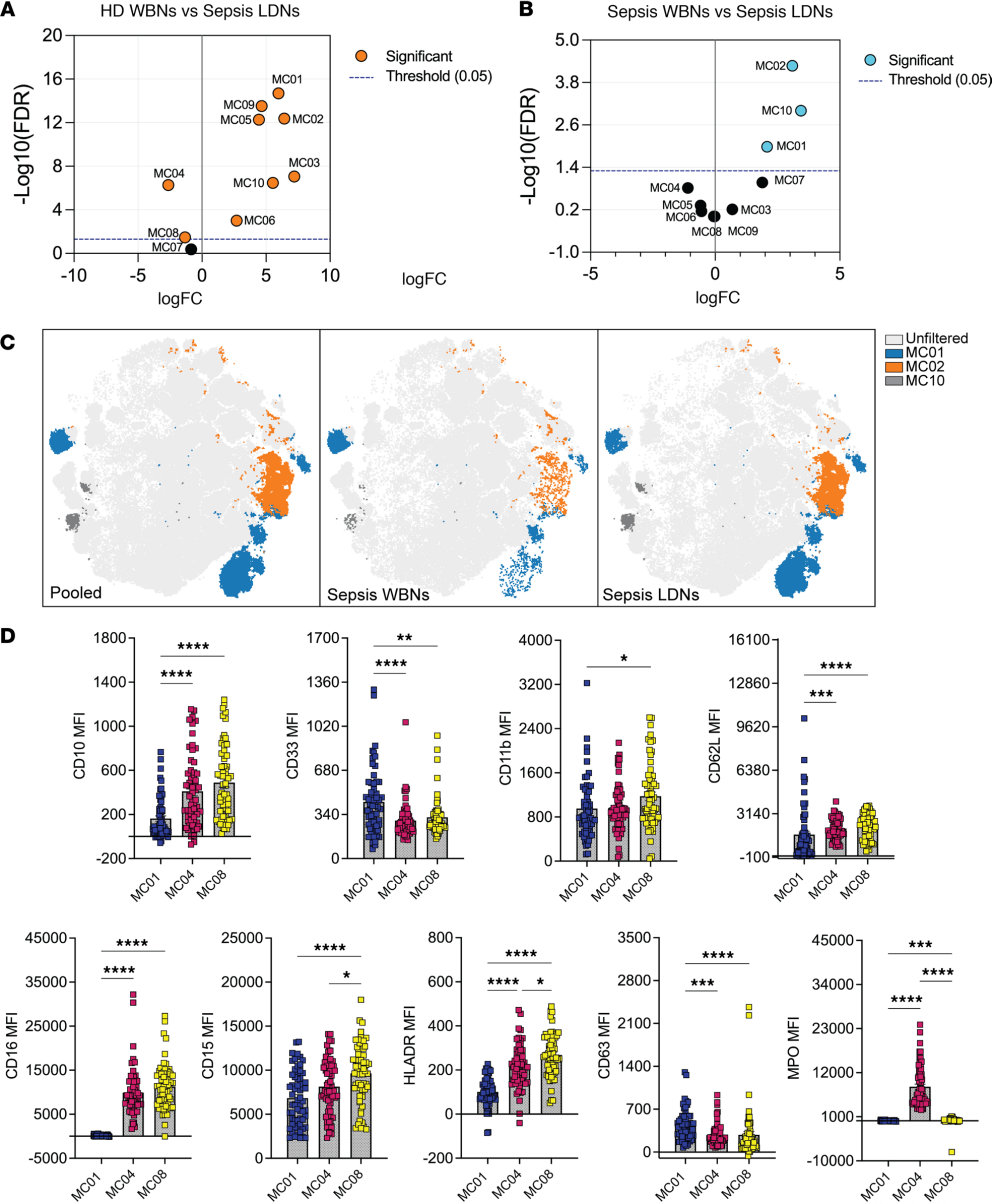
Characterization of sepsis LDNs versus healthy donor and sepsis WBNs. (**A**) Volcano plot comparing the logFC versus log10(FDR) comparing healthy donor WBNs and sepsis LDNs. Orange dots represent MCs that are statistically different between healthy donor WBNs and sepsis LDNs. (**B**) Volcano plot comparing the logFC versus log10(FDR) comparing sepsis WBNs and LDNs. Blue dots represent MCs that are statistically different between sepsis WBNs and LDNs. (**C**) optSNE plot representing MC01, MC02, and MC10 across pooled samples and sepsis WBNs and LDNs. (**D**) Bar graphs comparing relative expression of individual features across MC01, MC04, and MC08. Data are represented as means ± SEM. Significance was determined using a 1-way ANOVA with a Tukey’s multiple comparisons test or a Kruskal-Wallis with a Dunn’s multiple comparison test and set at *P* < 0.05*, *P* < 0.01**, *P* < 0.001***, *P* < 0.0001****. logFC, log fold-change; FDR, false discovery rate; optSNE, optimized t-distributed stochastic neighbor embedding; MC, metacluster; WBNs, whole blood neutrophils; HD, healthy donor; Sep, sepsis; LDNs, low-density neutrophils; SEM, standard error of the mean.

A similar distribution of MCs were increased in sepsis LDNs compared with HD WBNs, including MC09 (logFC 4.66, FDR < 0.01), MC05 (logFC 4.44, FDR < 0.01), MC01 (logFC 5.98, FDR < 0.01), MC02 (logFC 6.43, FDR < 0.01), MC03 (logFC 7.21, FDR < 0.01), MC10 (logFC 5.53, FDR < 0.01), and MC06 (logFC 2.71, FDR < 0.01) (Figure 3A, Supplemental Figure 4, and Supplemental Table 4). While the predominant MCs in sepsis WBNs and sepsis LDNs were similar (MC05 and MC09), MC01 (logFC 2.08, FDR < 0.01), MC02 (logFC 3.09, FDR < 0.01), and MC10 (logFC 3.44, FDR < 0.01) were significantly higher in LDNs compared with sepsis WBNs and appeared to be predominantly composed of LDNs (Figure 3, B and C; Supplemental Figure 4; and Supplemental Table 5). Compared with the HD populations (MC04 and MC08), MC01 had decreased CD10 (*P* < 0.0001), CD16 (*P* < 0.0001), CD62L (*P* < 0.001), and HLA-DR (*P* < 0.0001) and increased CD33 (*P* < 0.0001) and CD63 (*P* < 0.001) (Figure 3D). Similarly, MC02 demonstrated decreased CD10 (*P* < 0.0001), CD11b (*P* < 0.0001), CD62L (*P* < 0.0001), CD16 (*P* < 0.0001), and HLA-DR (*P* < 0.0001) compared with MC04 and MC08, while MC02 and MC04 had higher MPO than MC08 (*P* < 0.0001) (Supplemental Figure 6).

We next compared the predominant sepsis WBN MCs (MC05 and MC09) with the LDN-specific clusters (MC01 and MC02) (Supplemental Figure 7). Compared with MC05 and MC09, MC01 had increased CD33 (*P* < 0.001) and CD63 (*P* < 0.01) and decreased MPO (*P* < 0.0001), CD62L (*P* < 0.01), and CD16 (*P* < 0.0001) (Supplemental Figure 7A), while MC02 had decreased CD10 (*P* < 0.0001), CD62L (*P* < 0.0001), CD16 (*P* < 0.0001), CD11b (*P* < 0.0001), HLA-DR (*P* < 0.01), and increased MPO (*P* < 0.0001) (Supplemental Figure 7B). In totality, these population-level differences suggest that sepsis neutrophils, both WBNs and LDNs, have an immature (lower CD10 and higher CD33) and activated phenotype with high levels of degranulation markers, compared with the healthy populations, while LDNs are more immature and activated than WBNs.

### LDNs consist of phenotypically heterogenous populations of neutrophils.

**Figure 4:**
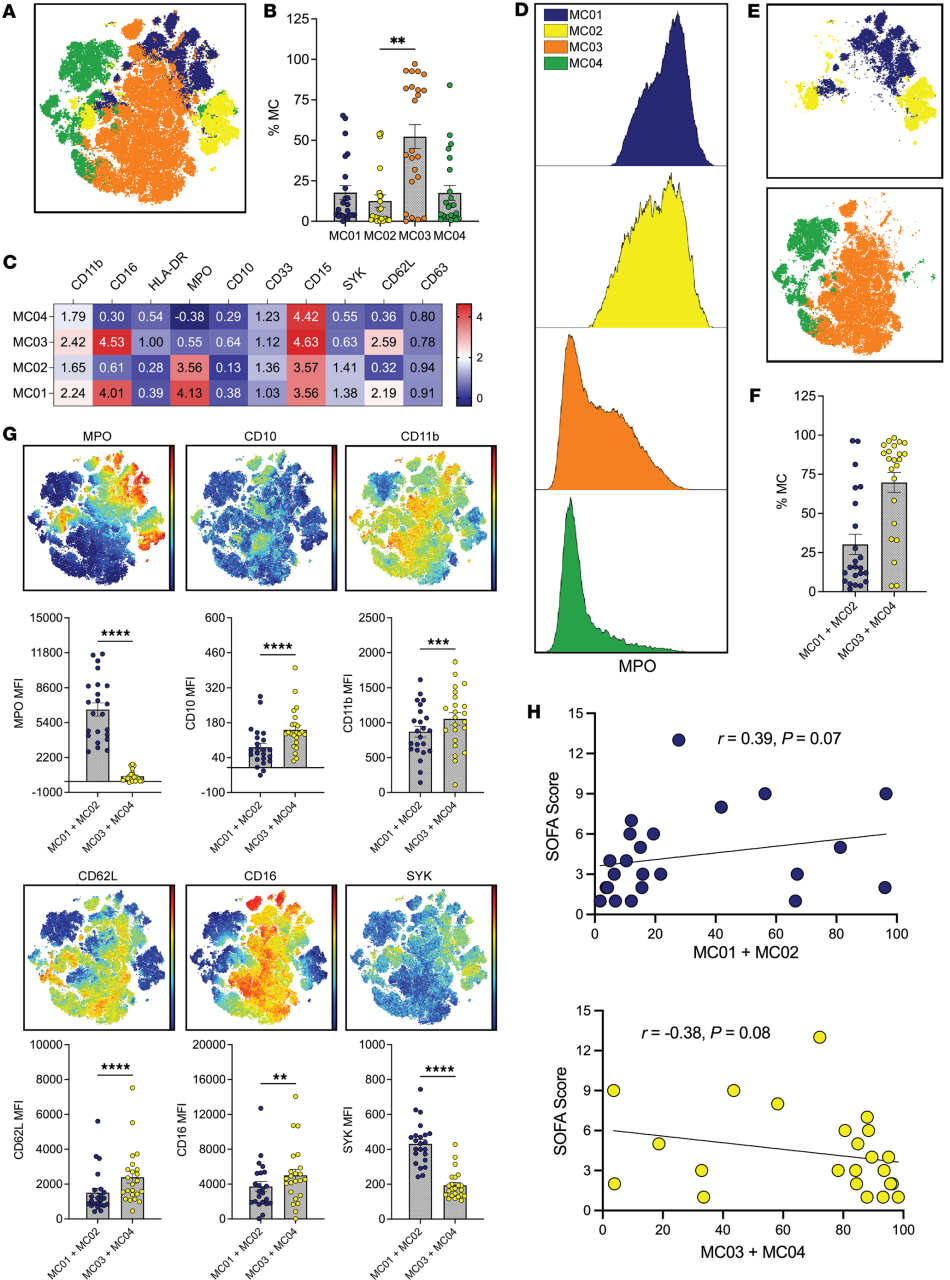
Characterization of LDNs. (**A**) optSNE plot of flow cytometry data overlaid with FlowSOM MCs for sepsis LDNs. (**B**) Abundance plot demonstrating the percentage of each MC. (**C**) Heatmap representing the relative expression of each extracellular and intracellular feature between MCs. (**D**) Histograms representing the relative expression of MPO across each MC. (**E**) optSNE plot of flow cytometry data overlaid with FlowSOM split by LDN-MC01 and LDN-MC02 combined and LDN-MC03 and LDN-MC04 combined. (**F**) Abundance plot demonstrating the percentage of MC01 and MC02 combined compared with MC03 and MC04 combined. (**G**) optSNE and bar graphs representing the expression of features in LDN-MC01 and LDN-MC02 compared with LDN-MC03 and LDN-MC04. (**H**) Correlation of LDN-MC01 and LDN-MC02 summed and LDN-MC03 and LDN-MC04 summed with SOFA score. Data are represented as means ± SEM. Significance was determined using a Friedman test, a paired *t* test, or a Wilcoxon test and set at *P* < 0.01**, *P* < 0.001***, *P* < 0.0001****. optSNE, optimized t-distributed stochastic neighbor embedding; MC, metacluster; MPO, myeloperoxidase; SEM, standard error of the mean.

To further characterize LDNs, we utilized dimensional reduction with clustering by FlowSOM to assess heterogeneity within sepsis LDNs and identified 4 MCs (Figure 4, A and B). LDN-MC03 accounted for the highest percentage of neutrophils at 52.3% followed by LDN-MC01 (17.7%), LDN-MC04 (17.5%), and LDN-MC02 (12.4%) (Figure 4B). A distinguishing feature between the LDN-MCs was the differential expression of MPO, which was high in LDN-MC01 and LDN-MC02 and low in LDN-MC03 and LDN-MC04, indicative of one population of LDNs that may have degranulated and another that retained its granules (Figure 4, C–E). We therefore compared the average MFI of LDN-MC01 and LDN-MC02 combined and LDN-MC03 and LDN-MC04 for each intracellular and extracellular feature to determine differential expression across the low-MPO LDN (LDN-MC03 and LDN-MC04) and high-MPO LDN (LDN-MC01 and LDN-MC02) populations (Figure 4, F and G). The high-MPO populations had a significantly higher average MPO MFI (*P* < 0.001) (Figure 4G). Furthermore, the MPO-low population had higher CD10 compared with the MPO-high populations, suggesting the MPO-low populations are mature neutrophils, while the MPO-high populations are more immature (Figure 4G). Comparing the additional features, we found that the MPO-low population had higher CD11b (*P* < 0.001), CD62L (*P* < 0.001), CD16 (*P* < 0.001), and HLA-DR (*P* < 0.001) (Figure 4G, Supplemental Figure 8). Interestingly, we found that the immature MPO-high populations trended to have a positive correlation with SOFA score, and a similar negative correlation was identified in the mature MPO-low population (*P* = 0.07, *P* = 0.08) (Figure 4H).

### SYK expression is increased in WBNs and LDNs of patients with sepsis.

**Figure 5:**
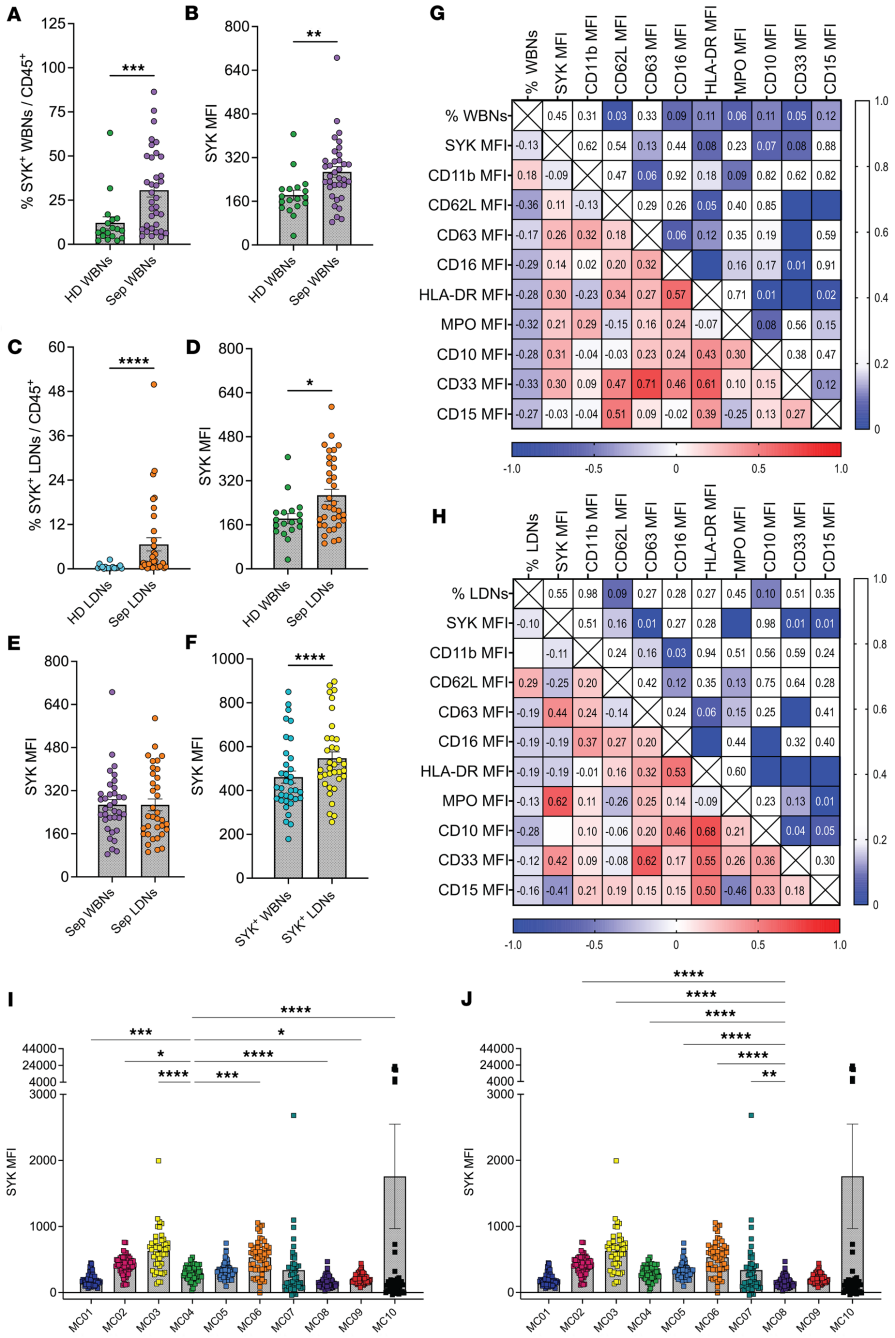
Characterization of SYK expression in WBNs and LDNs. (**A**) Bar graph presenting the percentage of SYK^+^ WBNs as a proportion of CD45 and (**B**) total SYK expression in WBNs across healthy donors and sepsis patients. (**C**) Bar graph presenting the percentage of SYK^+^ LDNs as proportion of CD45 and (**D**) total SYK expression in LDNs across healthy donor and sepsis patients. (**E**) Bar graph representing the SYK expression in WBNs versus LDNs and (**F**) SYK expression in SYK^+^ WBNs versus SYK^+^ LDNs in sepsis patients. (**G**) Heatmap representing the correlation matrix of each feature among WBNs and (**H**) LDNs. (**I**) Bar graphs comparing relative expression of SYK across all 10 metaclusters compared with MC04 (**J**) compared with MC08, the predominate healthy donor populations. Data are represented as means ± SEM. Significance was determined using a Mann-Whitney test, a paired *t* test, a Wilcoxon test, or a Kruskal-Wallis test with Dunn’s multiple comparisons test and set at *P* < 0.05*, *P* < 0.01**, *P* < 0.001***, *P* < 0.0001****. SYK, spleen tyrosine kinase; WBNs, whole blood neutrophils; HD, healthy donor; Sep, sepsis; MFI, mean fluorescence intensity; LDNs, low-density neutrophils; MC, metacluster; SEM, standard error of the mean.

We next sought to determine if SYK expression is increased in WBNs and LDNs of patients with sepsis. Comparing patient with HD WBNs, we found that patients with sepsis had a higher percentage of SYK^+^ WBNs (30.6% vs. 11.7%, *P* < 0.001) and total SYK expression (*P* < 0.01) (Figure 5, A and B). Similarly, comparing HD LDNs with sepsis LDNs, patients with sepsis had a higher percentage of SYK^+^ LDNs (6.6% vs. 0.6%, *P* < 0.001), and total SYK expression was higher in sepsis LDNs compared with both HD WBNs (*P* < 0.05) and HD LDNs (*P* < 0.05) (Figure 5, C and D, and Supplemental Figure 3). While there was no difference in total SYK expression between sepsis WBNs and LDNs (Figure 5E), there was higher SYK expression in sepsis SYK^+^ LDNs versus sepsis SYK^+^ WBNs (*P* < 0.0001) (Figure 5F). While SYK expression in WBNs did not have a correlation with any other features, SYK expression in LDNs had a positive correlation with CD33 (*r* = 0.42; *P* < 0.01), CD63 (*r* = 0.44; *P* < 0.01), and MPO (*r* = 0.62; *P* < 0.01) and negatively correlated with CD15 (*r* = –0.41; *P* < 0.01) (Figure 5, G and H). At the population level, when comparing with MC04, a population of neutrophils primarily made of HDs, the LDN populations of MC03 (*P* < 0.0001), MC02 (*P* < 0.05), and MC10 (*P* < 0.0001), along with the sepsis WBN and LDN population MC06 (*P* < 0.001), expressed statistically higher SYK (Figure 5I). Similarly, when comparing with MC08, the other predominate HD population, MC02 (*P* < 0.0001), MC03 (*P* < 0.0001), MC04 (*P* < 0.0001), MC05 (*P* < 0.0001), MC06 (*P* < 0.0001), and MC07 (*P* < 0.01) all expressed higher levels of SYK (Figure 5J).

### SYK^+^ WBNs and LDNs are more immature and activated than SYK^–^ WBNs and LDNs in patients with sepsis.

**Figure 6:**
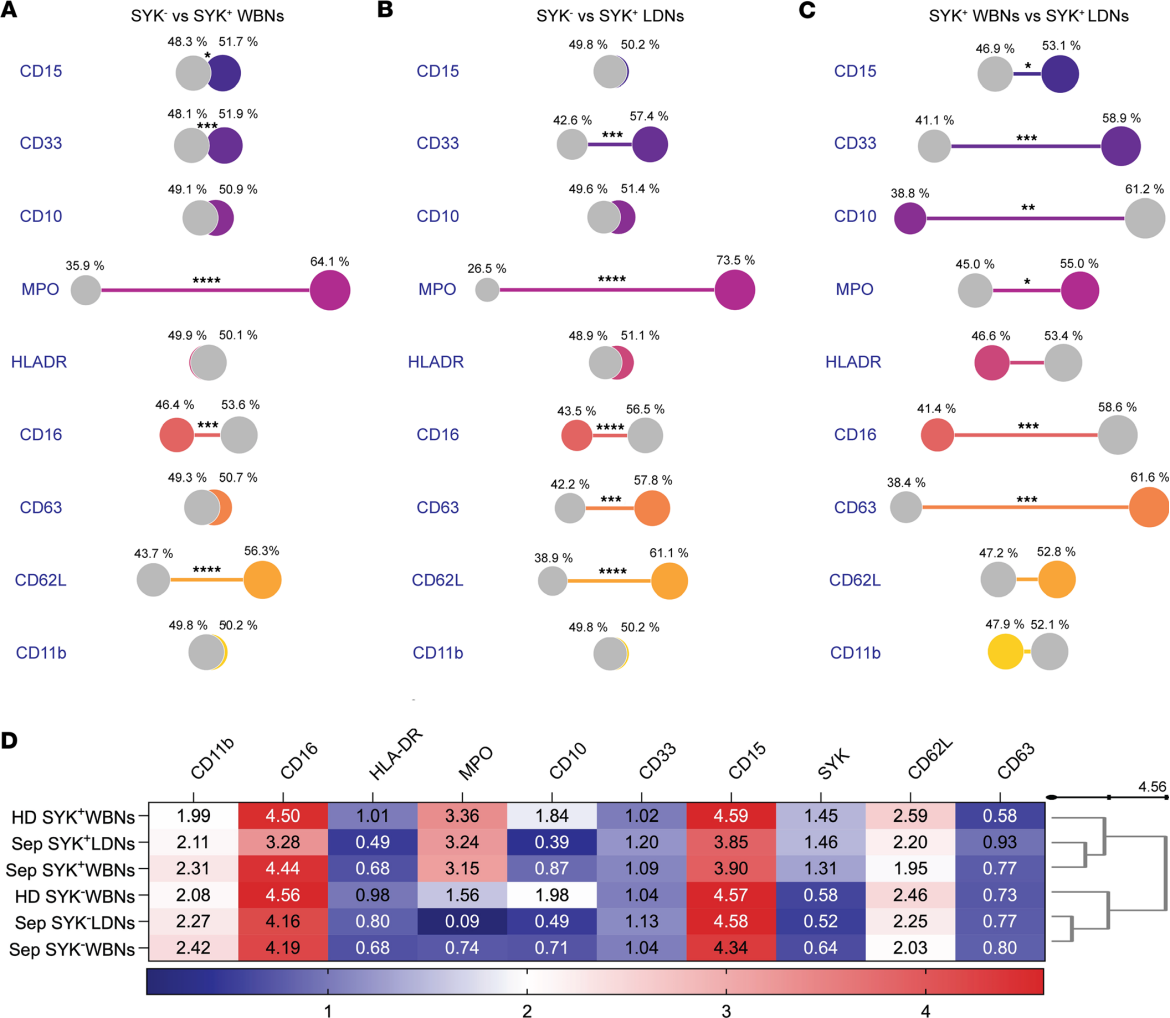
Characterization of SYK^+^ and SYK^–^ neutrophil subsets from patients with sepsis. (**A**) Dumbbell plot comparing the percentage of expression of each extracellular and intracellular feature across SYK^–^ WBNs (gray) and SYK^+^ WBNs (color), (**B**) SYK^–^ LDNs (gray) and SYK^+^ LDNs (color), and (**C**) SYK^+^ WBNs (gray) and SYK^+^ LDNs (color). The size of the circle represents the magnitude of the percentage of each extracellular and intracellular feature, and the distance between the circles represents the percentage difference between each feature for the neutrophil subsets being compared. (**D**) Heatmap representing the relative expression of each extracellular and intracellular feature between healthy donor SYK^+^ WBNs, healthy donor SYK^–^ WBNs, sepsis SYK^+^ WBNs, sepsis SYK^–^ WBNs, sepsis SYK^+^ LDNs, and sepsis SYK^–^ LDNs. Data are represented as means ± SEM. Significance was determined using a paired *t* test or a Wilcoxon test and set at *P* < 0.05*, *P* < 0.01**, *P* < 0.001***, *P* < 0.0001****. SYK, spleen tyrosine kinase; HD, healthy donor; Sep, sepsis; WBNs, whole blood neutrophils; LDNs, low-density neutrophils; SEM, standard error of the mean.

We next compared expression of each feature across SYK^+^ and SYK^–^ neutrophil populations among the patients with sepsis. Comparing SYK^+^ WBNs with SYK^–^ WBNs, we found that SYK^+^ WBNs expressed a higher percentage of CD15 (*P* < 0.05), CD33 (*P* < 0.001), CD62L (*P* < 0.0001), and MPO (*P* < 0.0001) and lower CD16 (*P* < 0.001) (Figure 6A). Among the LDNs, SYK^+^ LDNs expressed a higher percentage of CD33 (*P* < 0.001), CD62L (*P* < 0.0001), MPO (*P* < 0.0001), and CD63 (*P* < 0.001) and lower CD16 (*P* < 0.0001) (Figure 6B). When comparing SYK^+^ WBNs and SYK^+^ LDNs, we observed that SYK^+^ LDNs expressed a higher percentage of CD15 (*P* < 0.05), CD33 (*P* < 0.001), MPO (*P* < 0.05), and CD63 (*P* < 0.001) and lower CD10 (*P* < 0.01) and CD16 (*P* < 0.001) (Figure 6C). We further assessed the total expression (MFI) of each of these features across SYK^+^ WBNs to SYK^–^ WBNs, SYK^+^ LDNs to SYK^–^ LDNs, and SYK^+^ WBNs to SYK^+^ LDNs and found that differential expression produced similar results when comparing percentages (Figure 6, A–C), except for higher CD15 in SYK^+^ WBNs versus SYK^–^ WBNs, which did not reach statistical significance (Supplemental Figure 9, A–C).

Last, we compared SYK^+^ and SYK^–^ neutrophils at the population level across HD WBNs and sepsis WBNs and LDNs (Figure 6D). The 3 SYK-positive populations clustered most closely together with a second nodal clustering of SYK^+^ sepsis WBNs and LDNs (Figure 6D). The feature that appeared to discriminate most strongly between SYK^+^ and SYK^–^ populations was MPO, which was elevated in all the SYK^+^ populations. Comparing SYK^+^ WBNs and SYK^+^ LDNs revealed that SYK^+^ LDNs have increased CD33 and CD62L and decreased CD10 and CD16 (Figure 6D). Additionally, in the dimensional reduction analysis of pure LDNs, the immature populations with higher MPO expression (LDN-MC01 and LDN-MC02) expressed higher SYK (*P* < 0.0001) than the mature populations, providing further evidence that SYK is associated with MPO expression and may be involved in neutrophil maturation (Figure 4G).

### LDNs and SYK expression correlate with soluble biomarkers.

**Figure 7:**
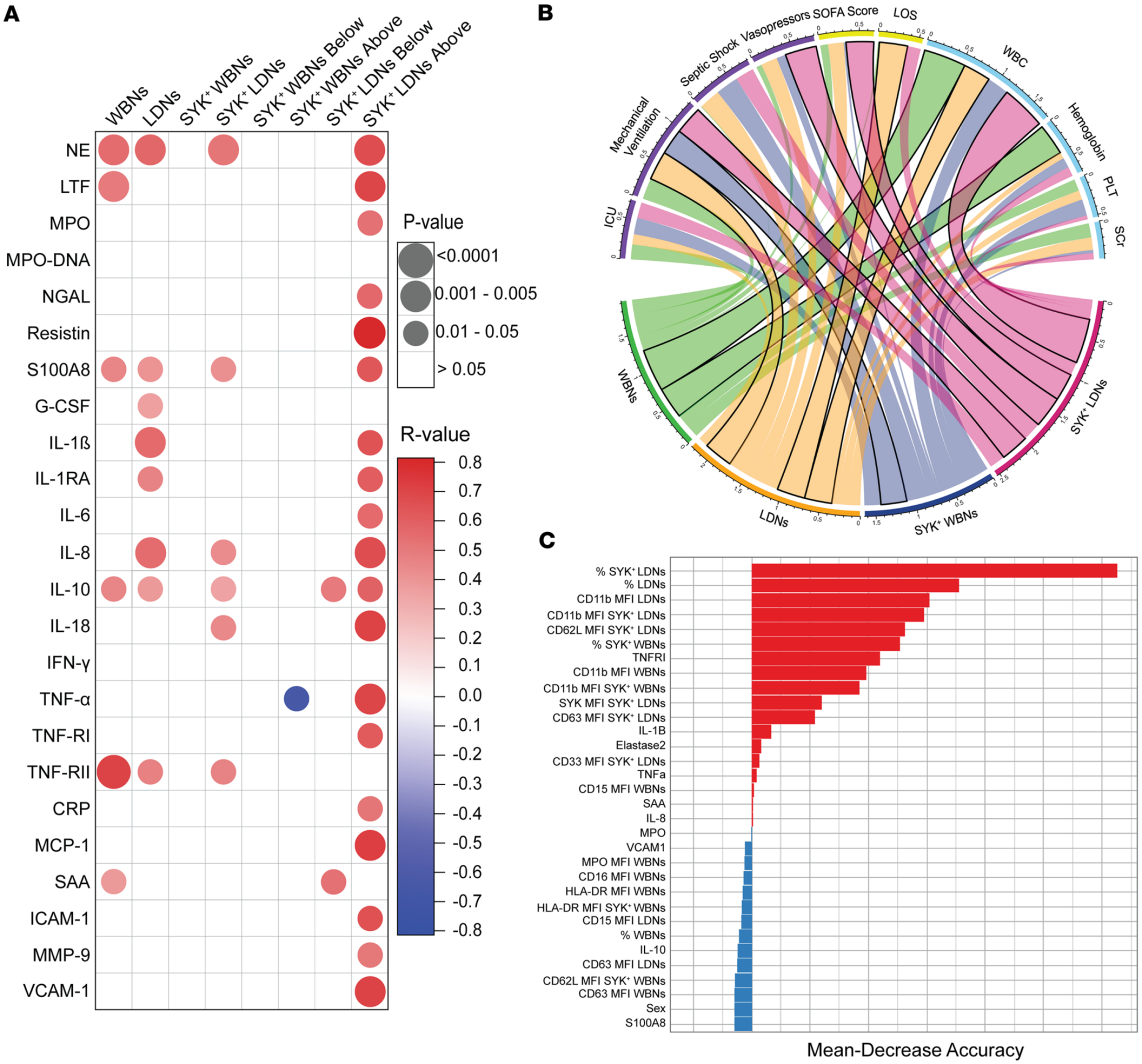
Correlations of WBNs, LDNs, SYK^+^ WBNs, and SYK^+^ LDNs with soluble biomarkers and clinical outcomes. (**A**) Bubble plot demonstrating the correlations of WBNs, LDNs, SYK^+^ WBNs, and SYK^+^ LDNs with soluble biomarkers. The color of the circle represents the strength of the correlations (*r* value), and size represents the statistical significance (*P* value). (**B**) Circos plot visualizing the correlation of the lower outer ring flow cytometry data quantifying WBNs, LDNs, SYK^+^ WBNs, and SYK^+^ LDNs with upper outer ring clinical outcomes and laboratory values. The ribbon width represents the strength of the association between 2 variables, with those that are statistically significant outlined in black. (**C**) Feature importance plot of clinical data, neutrophil populations, and biomarkers that distinguish patients on mechanical ventilation. The *x* axis is the mean accuracy of each variable in predicting mechanical ventilation. Red indicates a positive association and blue a negative association. Associations for Circos plot were performed with Spearman’s correlations for continuous variables and point-biserial for dichotomous outcomes. SYK, spleen tyrosine kinase; WBNs, whole blood neutrophils; LDNs, low-density neutrophils; MFI, mean fluorescence intensity; ICU, intensive care unit; SOFA, sequential organ failure assessment; LOS, length of stay; WBC, white blood cell; PLT, platelet count; SCr, serum creatinine; NE, elastase-2; LTF, lactoferrin; SAA, serum amyloid A.

The proportion of WBNs had a positive correlation with the concentration of soluble elastase-2 (*r* = 0.53, *P* < 0.01), lactoferrin (*r* = 0.48, *P* < 0.01), S100A8/MRP8 (*r* = 0.44, *P* < 0.01), IL-10 (*r* = 0.44, *P* < 0.01), TNF-RII (*r* = 0.69, *P* < 0.0001), and SAA (*r* = 0.38, *P* < 0.05) (Figure 7A and Supplemental Table 6). Similarly, the proportion of LDNs correlated with elastase-2 (*r* = 0.56, *P* < 0.001), S100A8/MRP8 (*r* = 0.39, *P* < 0.05), IL-1RA (*r* = 0.45, *P* < 0.01), IL-1β (*r* = 0.54, *P* < 0.001), IL-8 (*r* = 0.53, *P* < 0.01), IL-10 (*r* = 0.37, *P* < 0.05), TNF-RII (*r* = 0.46, *P* < 0.01), and G-CSF (*r* = 0.35, *P* < 0.05) (Figure 7A and Supplemental Table 6). We next looked at correlations of each biomarker with SYK^+^ WBNs and SYK^+^ LDNs. While we did not observe any correlations with SYK^+^ WBNs, we found positive correlations between SYK^+^ LDNs and elastase-2 (*r* = 0.50, *P* < 0.01), S100A8/MRP8 (*r* = 0.40, *P* < 0.05), IL-8 (*r* = 0.42, *P* < 0.01), IL-10 (*r* = 0.34, *P* < 0.05), TNF-RII (*r* = 0.46, *P* < 0.01), and IL-18 (*r* = 0.43, *P* < 0.01) (Figure 7A and Supplemental Table 6).

We next wanted to determine if each of these biomarkers had a stronger correlation in those patients who expressed high levels of SYK compared with lower levels. We found that when we dichotomized WBNs, LDNs, and their respective SYK expression into those with high SYK (above the median) and low SYK (below the median), there were very few biomarkers associated with higher levels of WBNs, LDNs, and SYK^+^ WBNs (Figure 7A and Supplemental Table 7). However, among the SYK^+^ LDNs in the above-median group there were significant positive correlations with 19/24 biomarkers, including the neutrophil markers elastase-2, lactoferrin, MPO, neutrophil gelatinase-associated lipocalin (NGAL), resistin, and S100A8; the inflammatory markers IL-1β, IL-1 receptor antagonist (IL-1RA), IL-6, IL-8, IL-10, IL-18, TNF-α, TNF receptor 1 (TNF-RI), C-reactive protein (CRP), and monocyte chemoattractant protein-1 (MCP-1); and the endothelial makers intercellular adhesion molecule 1 (ICAM-1), matrix metalloproteinase 9 (MMP9), and vascular cell adhesion molecule 1 (VCAM-1) (Figure 7A and Supplemental Table 7).

### LDNs and SYK expression correlate with disease severity and white blood cell count.

We next hypothesized that neutrophil counts and SYK expression would associate with disease severity. We did not find any correlations between WBNs and disease severity; however, the percentage of LDNs associated with the need for mechanical ventilation (*r_pb_* = 0.37, *P* < 0.05), length of stay (*r* = 0.36, *P* < 0.05), and white blood cell (WBC) count (*r* = 0.35, *P* < 0.05) (Figure 7B). When evaluating SYK^+^ populations we observed that the percentage of SYK^+^ WBNs had an association with the need for mechanical ventilation (*r* = 0.33, *P* = 0.05), while SYK^+^ LDNs had an association with mechanical ventilation (*r_pb_* = 0.36, *P* < 0.05), vasopressors (*r_pb_* = 0.40, *P* < 0.05), SOFA score (*r* = 0.35, *P* < 0.05), and WBC (*r* = 0.57, *P* < 0.001) (Figure 7B).

Given these associations we then sought to determine if any demographic variables, soluble biomarkers, or neutrophil populations could predict disease severity. To evaluate this, we performed a random forest model to identify the features of greatest importance between patients who were on mechanical ventilation and those who were not. The features that most strongly predicted need for mechanical ventilation were the percentages of SYK^+^ LDNs and total LDNs (Figure 7C). Confirming the importance of SYK in predicting the need for mechanical ventilation, the population of WBNs that had the strongest prediction was SYK^+^ WBNs. Additionally, CD11b MFI was found to be an important feature on LDNs, and SYK^+^ LDNs and WBNs and SYK^+^ WBNs.

Because SYK levels and LDN proportions are not readily available at the bedside, we next wanted to determine if LDNs and SYK expression associate with any clinical or laboratory variable easily identifiable at the bedside. To do this, we stratified patients into those above and below the median for the proportion of LDNs and SYK^+^ LDNs and compared clinical variables of each group. We found that many clinical variables of disease severity were higher in the above-median group for LDNs and SYK^+^ LDNs. Specifically, for LDNs the above-median group had a higher number of patients with septic shock (*P* < 0.01), on mechanical ventilation (*P* < 0.05), while the WBC (*P* < 0.05) and length of stay (*P* < 0.05) were also higher in the above-median group (Supplemental Table 8). Similarly, when comparing the above-median and below-median groups for SYK^+^ LDNs, there were a higher percentage of patients on mechanical ventilation (*P* < 0.05), higher WBC (*P* < 0.01), and longer length of stay (*P* < 0.05) in the above-median group (Supplemental Table 9).

### Responsiveness of normal-density neutrophils (NDNs) and LDNs and SYK^+^ and SYK^–^ subsets following lipopolysaccharide (LPS) stimulation.

**Figure 8:**
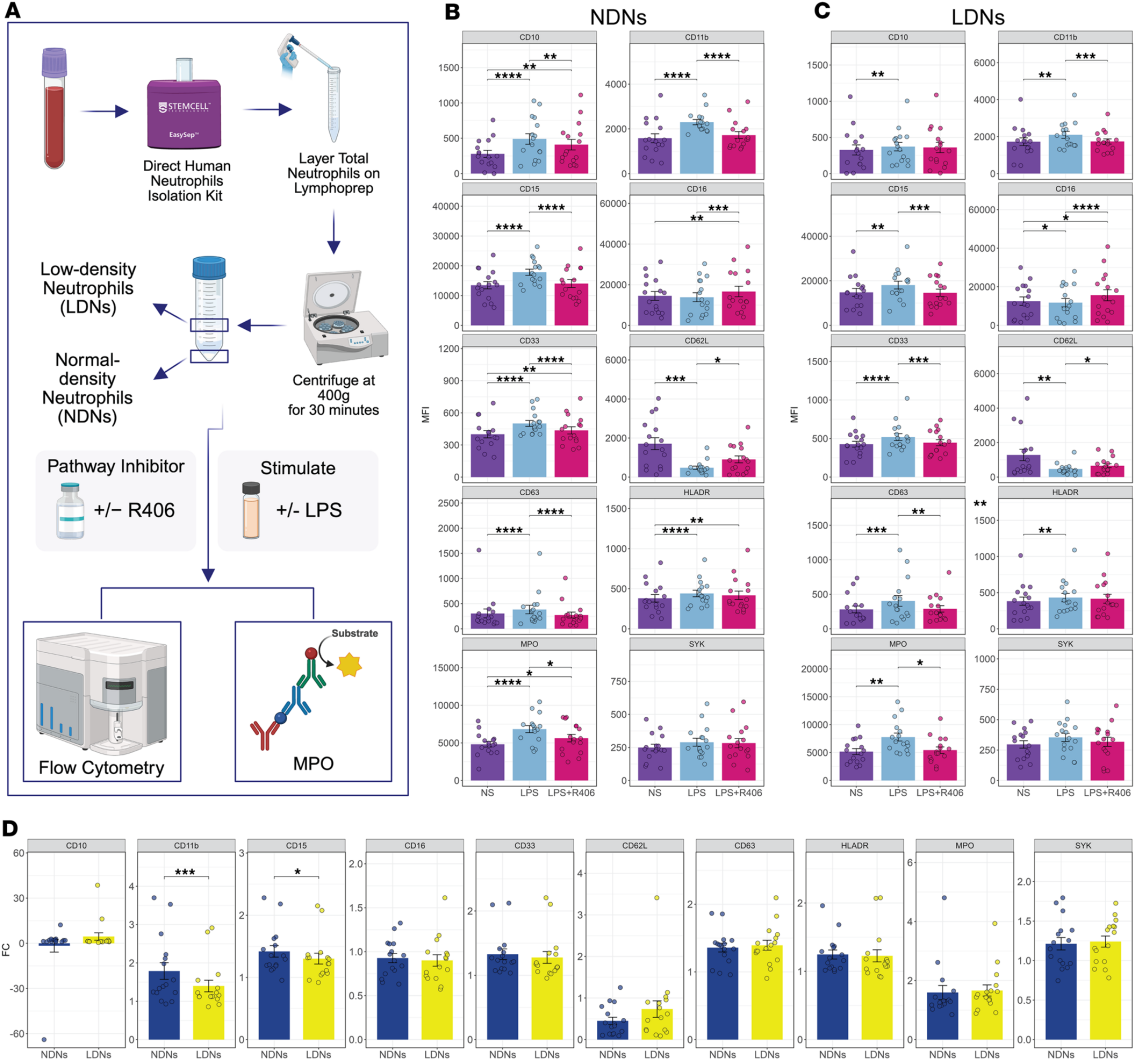
Functional assessment of NDNs and LDNs following LPS stimulation and SYK inhibition. (**A**) Schematic representation of methods for stimulation of NDNs and LDNs from patients with sepsis (*N* = 16). (**B**) Bar graphs representing the MFI of each individual feature comparing nonstimulated (purple), LPS-stimulated (blue), and LPS stimulation following R406-treated (pink) NDNs. (**C**) Bar graphs representing the MFI of each individual feature comparing nonstimulated (purple), LPS-stimulated (blue), and LPS stimulation following R406-treated (pink) LDNs. (**D**) Bar graphs representing the fold-change in feature expression following LPS stimulation comparing NDNs (dark blue) and LDNs (yellow). Data are represented as means ± SEM. Significance was determined using a Conover’s post hoc test for Friedman’s test with a Bonferroni correction or a Wilcoxon test and set at *P* < 0.05*, *P* < 0.01**, *P* < 0.001***, *P* < 0.0001****. NDNs, normal-density neutrophils; LDNs, low-density neutrophils; LPS, lipopolysaccharide; NS, nonstimulated; MFI, mean fluorescence intensity; FC, fold-change; SEM, standard error of the mean.

**Figure 9:**
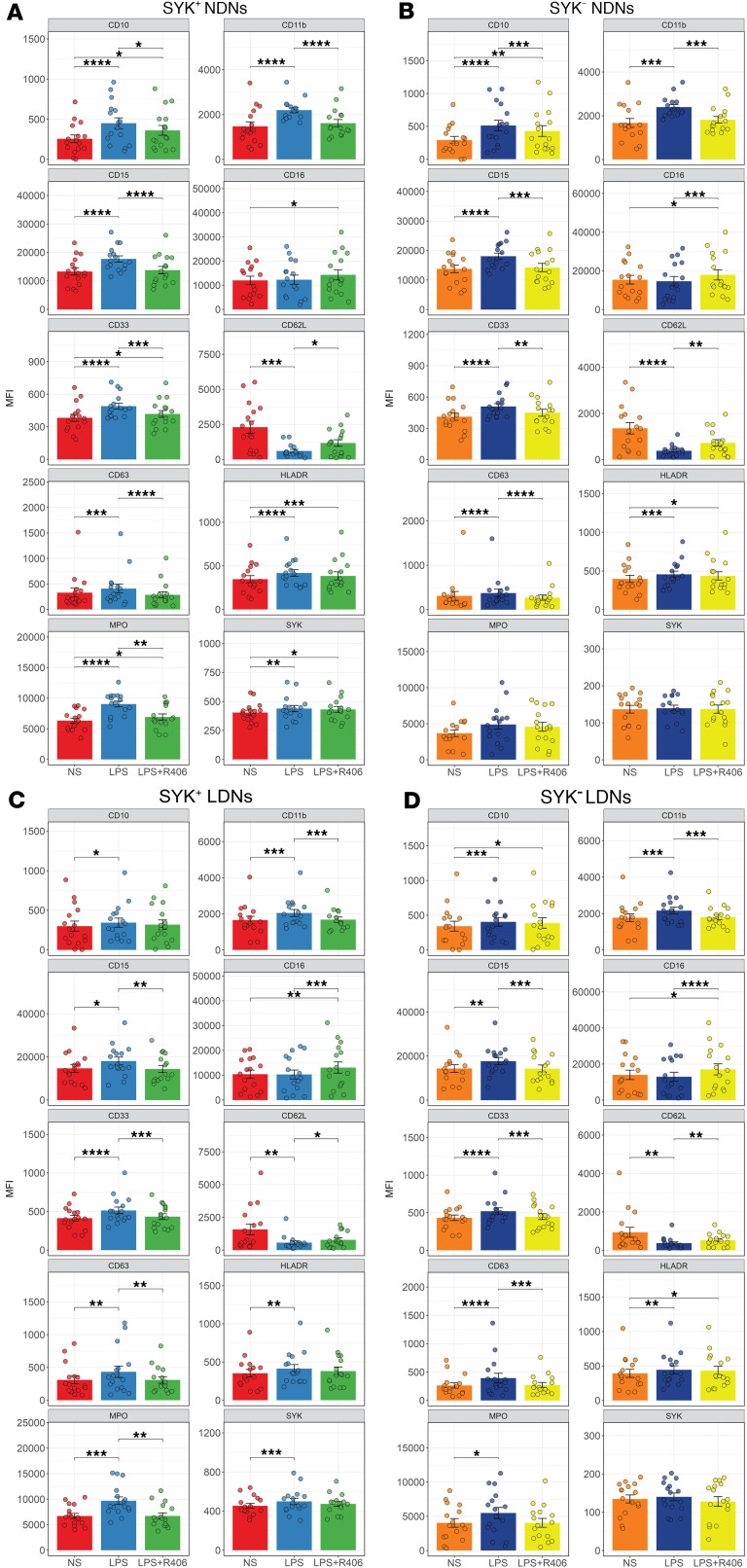
Functional assessment of SYK^+^ and SYK^–^ NDNs and SYK^+^ and SYK^–^ LDNs following LPS stimulation and SYK inhibition. (**A**) Bar graphs representing the MFI of each individual feature comparing nonstimulated (red), LPS-stimulated (blue), and LPS stimulation following R406-treated (green) SYK^+^ NDNs. (**B**) Bar graphs representing the MFI of each individual feature comparing nonstimulated (orange), LPS-stimulated (dark blue), and LPS stimulation following R406-treated (yellow) SYK^–^ NDNs. (**C**) Bar graphs representing the MFI of each individual feature comparing nonstimulated (red), LPS-stimulated (blue), and LPS stimulation following R406-treated (green) SYK^+^ LDNs. (**D**) Bar graphs representing the MFI of each individual feature comparing nonstimulated (orange), LPS-stimulated (dark blue), and LPS stimulation following R406-treated (yellow) SYK^–^ LDNs. Data are represented as means ± SEM. Significance was determined using a Conover’s post hoc test for Friedman’s test with a Bonferroni correction and set at *P* < 0.05*, *P* < 0.01**, *P* < 0.001***, *P* < 0.001***. NDNs, normal-density neutrophils; LDNs, low-density neutrophils; LPS, lipopolysaccharide; NS, nonstimulated; MFI, mean fluorescence intensity; SEM, standard error of the mean.

We next sought to characterize phenotypic changes in purified NDNs and LDNs’ features following LPS stimulation (Figure 8A). LPS stimulation led to statistically significant changes in all the evaluated features except for CD16 and SYK in NDNs and SYK in the LDN subsets (Figure 8, B and C). To determine if NDNs or LDNs are more responsive to LPS stimulation, we next compared the fold-change from baseline following stimulation across the 2 groups and found that LPS stimulation resulted in a statistically significant increase in CD11b (*P* < 0.001) and CD15 (*P* < 0.05) in NDNs compared with LDNs, suggesting that NDNs may have more reserve activation ability (Figure 8D). We similarly evaluated the responsiveness of the SYK^+^ and SYK^–^ subsets for both NDNs and LDNs and found that like the overall populations, there were statistically significant changes in most of the evaluated features (Figure 9, A–D). To assess if SYK^+^ or SYK^–^ NDNs and LDNs are more responsive to LPS, we again compared the fold-change from baseline following stimulation in these neutrophil subsets. We found that following stimulation SYK^+^ NDNs had statistically significant increases in CD11b (*P* < 0.05), CD16 (*P* < 0.0001), HLA-DR (*P* < 0.001), and CD33 (*P* < 0.05) compared with SYK^–^ NDNs (Supplemental Figure 10A), while SYK^+^ LDNs had higher CD16 (*P* < 0.01) and CD33 (*P* < 0.05) compared with SYK^–^ LDNs (Supplemental Figure 10B). Comparing SYK^+^ NDNs and SYK^+^ LDNs we found that LPS stimulation resulted in an increase in CD11b (*P* < 0.001) and CD10 (*P* < 0.05) in SYK^+^ NDNs compared with SYK^+^ LDNs (Supplemental Figure 10C). In totality, these data suggest that both NDNs and LDNs from patients with sepsis can be stimulated by LPS and that NDNs and SYK^+^ neutrophil subsets may be slightly more responsive than their LDN and SYK^–^ counterparts.

### R406 alters phenotypes of neutrophils from patients with sepsis following LPS stimulation.

After seeing differences in the responsiveness of NDNs and LDNs to LPS stimulation, we wanted to determine the impact of SYK inhibition with R406 on each feature. R406 was able to inhibit the relative changes in 8/10 features in LPS-stimulated NDNs, including CD10 (*P* < 0.01), CD11b (*P* < 0.0001), CD15 (*P* < 0.0001), CD16 (*P* < 0.001), CD33 (*P* < 0.0001), CD62L (*P* < 0.05), CD63 (*P* < 0.0001), and MPO (*P* < 0.05) (Figure 8B). Among the LDNs R406 was able to inhibit the relative changes in 7/10 evaluated features, including CD11b (*P* < 0.001), CD15 (*P* < 0.001), CD16 (*P* < 0.0001), CD33 (*P* < 0.001), CD62L (*P* < 0.05), CD63 (*P* < 0.01), and MPO (*P* < 0.05) (Figure 8C). Overall similar results were observed when evaluating the subsets of SYK^+^ and SYK^–^ NDNs and SYK^+^ and SYK^–^ LDNs (Figure 9, A–D).

### Impact of LPS stimulation and SYK inhibition on degranulation of sepsis neutrophils.

**Figure 10:**
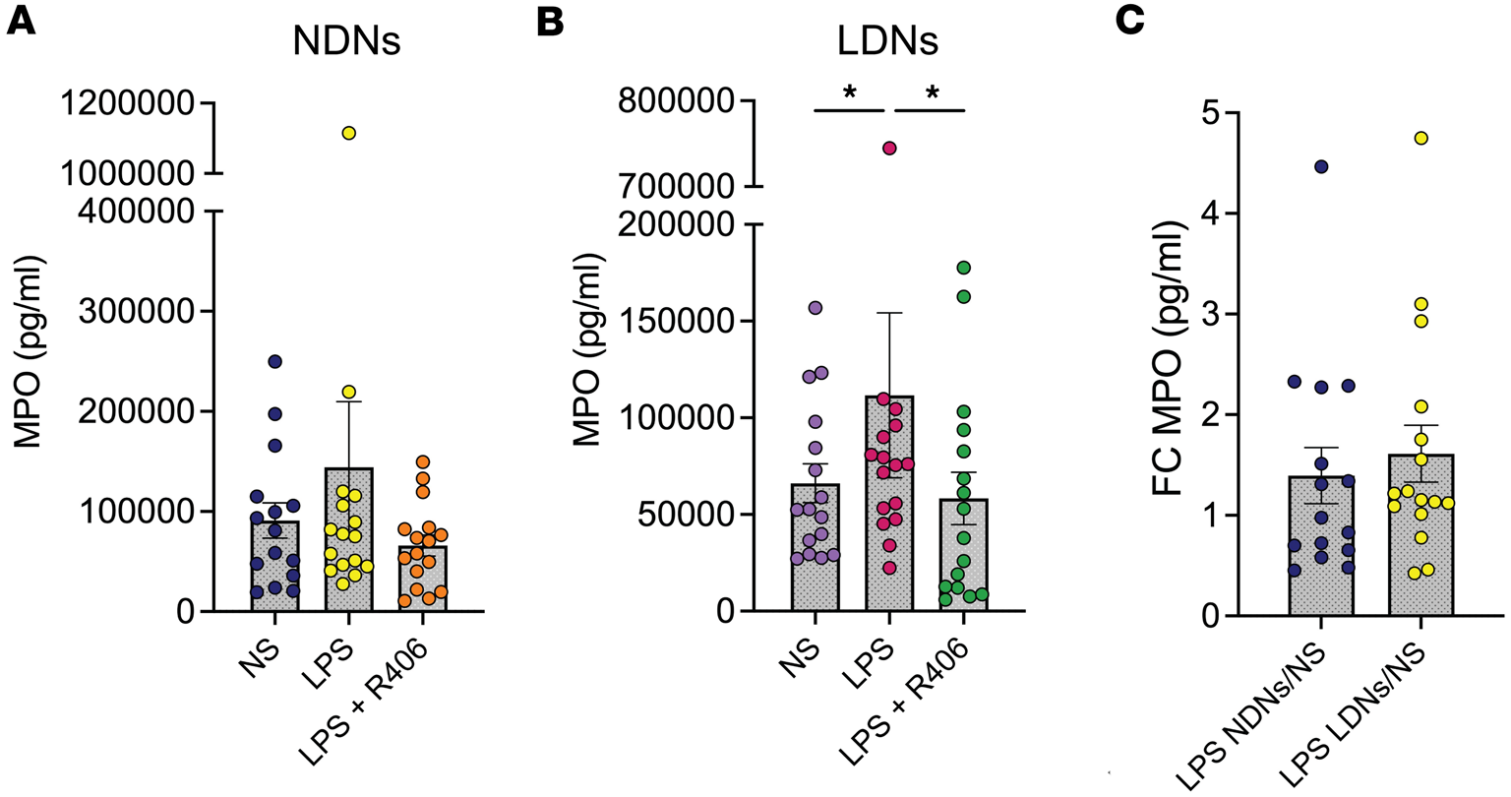
Functional assessment of degranulation among NDNs and LDNs and the impact of SYK inhibition. (**A**) Bar graph representing MPO levels among nonstimulated, LPS-stimulated, and LPS stimulation following R406-treated NDNs. (**B**) Bar graph representing MPO levels among nonstimulated, LPS-stimulated, and LPS stimulation following R406-treated LDNs. (**C**) Bar graph representing the fold-change in MPO release following LPS stimulation comparing NDNs and LDNs. Significance was determined using a Friedman’s test with a Dunn’s multiple comparisons test and set at *P < 0.05. NDNs, normal-density neutrophils; LDNs, low-density neutrophils; LPS, lipopolysaccharide; NS, nonstimulated; MPO, myeloperoxidase; SEM, standard error of the mean.

Degranulation is another important neutrophil function associated with poor outcomes in sepsis; therefore, we measured soluble MPO in cell supernatants following LPS stimulation. We found that LPS stimulation resulted in minimal increases in the amount of soluble MPO from NDNs, but there was a statistically significant increase in the amount of MPO from LDNs (*P* < 0.05) (Figure 10, A and B). While we did not see decreases in the release of MPO from NDNs with R406 SYK inhibition, we did observe a decrease in MPO in LDNs’ (*P* < 0.05) supernatants (Figure 10, A and B). Last, we compared the responsiveness of NDNs and LDNs and found no difference in the amount of MPO release between these subsets (Figure 10C).

## Discussion

Sepsis is associated with significant morbidity and mortality globally (23). To date the standard of care for patients with sepsis is primarily based on early appropriate antibiotics and supportive care for end-organ dysfunction, including fluids, vasopressors, and mechanical ventilation (24). Decades of research focused on unraveling sepsis pathogenesis and identifying host-directed therapies to modulate the immune response. However, despite substantial efforts there are no FDA-approved host-directed therapies targeting the dysregulated immune response that dominates sepsis (25). Herein, we identify that in human samples, LDNs are a heterogenous population of innate immune cells elevated during sepsis. In addition, SYK expression is increased in both WBNs and LDNs and correlates with disease severity, providing evidence that SYK inhibition might be a potentially novel therapeutic target during sepsis.

Neutrophil phenotypic and functional heterogeneity is becoming increasingly recognized (9). Recent evidence in chronic disease suggests that LDNs contribute to pathogenesis and associate with disease severity (11, 26). However, LDNs have been studied to a lesser extent in infections that cause critical illness including sepsis, though recently they have been implicated in the pathogenesis of COVID-19, where they are associated with disease severity, hypercoagulation, emergency granulopoiesis, and immunosuppressive states (27–31). In this study, we first compared sepsis LDNs and WBNs from HDs as a single population and found that sepsis LDNs expressed lower CD10 and higher CD33 consistent with an immature neutrophil generated during emergency granulopoiesis, along with higher CD63 and lower CD62L indicating an activated phenotype. Similarly, when comparing among patients with sepsis we found that sepsis LDNs were more immature (increased CD10 and CD33) and activated (increased CD15 and CD63) than their whole blood counterparts. These data are consistent with prior work in sepsis and chronic disease, such as systemic lupus erythematous, suggesting that LDNs, when evaluated as a single population, are primarily immature neutrophils (12, 32).

The origins of LDNs are still not fully understood. Despite original data suggesting that LDNs are primarily immature neutrophils, they are likely composed of heterogenous populations with different maturity status and end-effector functions, including a subset of neutrophils identified as polymorphonuclear myeloid-derived suppressor cells (PMN-MDSCs) (14, 26, 33). To date 2 main hypotheses prevail regarding the origin of LDNs including degranulation and NET release from mature neutrophils and the release of immature progenitor neutrophils from the bone marrow during emergency granulopoiesis (10). Our dimensional reduction analysis of LDNs identified 2 main populations of LDNs, one of which was characterized by high levels of MPO (LDN-MC01 and LDN-MC02) and another with low levels of MPO (LDN-MC03 and LDN-MC04). Congruent with both prior hypotheses, the high-MPO populations expressed low levels of CD10, indicating they are immature and may be produced in response to emergency granulopoiesis, while the low-MPO population expressed high levels of CD10, indicating mature populations of neutrophils that may have degranulated. Interestingly, while the mature population appeared to have the lowest levels of MPO likely contributing to circulating MPO levels, the immature high-MPO populations appeared to more strongly associate with disease severity. These data support prior findings in our nonhuman primate model and are the first to our knowledge in humans to link immature LDNs in sepsis with MPO expression and disease severity (11).

Outside of the characterization of PMN-MDSCs, which are isolated from PBMC fractions following density gradient centrifugation and functionally use arginase and H_2_O_2_ to prevent the proliferation and CD3 zeta-chain expression of T cells, the functional role of other LDN subsets, including their ability to perform important end-effector functions, is incompletely understood (33). In trauma, LDNs are suggested to have an exhausted phenotype based on cell surface marker expression, and in sepsis LDNs have been shown to have decreased chemotactic and phagocytic capabilities (12, 34). Our mechanistic experiments stimulating sepsis NDNs and LDNs with LPS found that both neutrophil populations were responsive to LPS across the majority of the tested features and MPO release. However, there were minimal differences when comparing between NDNs and LDNs, except CD11b and CD15, which were upregulated to a greater degree in sepsis NDNs. We found similar results when comparing each SYK^+^ and SYK^–^ population across LPS-stimulated NDNs and LDNs.

SYK inhibition was first tested in humans with critical illness as a therapeutic target in COVID-19, where it was found to be safe and mechanistically to impact the proportion of circulating LDNs and overall neutrophil activation (15, 17). Mouse models of LPS-induced acute lung and kidney injury have established early evidence that SYK inhibition may be beneficial in sepsis (35, 36). Building on work done in COVID-19 and in small animal models of sepsis, our group identified that SYK is elevated in WBNs and LDNs in a nonhuman primate model of bacterial sepsis and that SYK expression in both the WBNs and LDNs associated with end-organ dysfunction, including renal failure and coagulopathy (21). In this current study, we determined that SYK expression is increased in WBNs and LDNs of human patients with sepsis. We noted that there was a strong correlation with the percentage of SYK^+^ LDNs, with many soluble biomarkers associated with neutrophil activation and poor outcomes in sepsis including elastase-2, lactoferrin, MPO, NGAL, resistin, S100A8, and IL-8 (37–41). Furthermore, the percentage of SYK^+^ LDNs associated with SOFA score, along with the need for mechanical ventilation and vasopressor support. These data provide further evidence that SYK expression associates with disease severity and that SYK expression may be a critical mediator of sepsis pathogenesis.

Prior in vitro work has tested the impact of SYK inhibition with R406, the active component of the FDA-approved SYK inhibitor fostamatinib, on many end-effector functions of neutrophils associated with detrimental outcomes in sepsis following stimulation of HD neutrophils with LPS (22). SYK inhibition was found to inhibit the release of the neutrophil granule contents, including MPO, lactoferrin, and MMP9; the release of ROS and NETs; and the adhesion of neutrophils to endothelial cells. Importantly, other important neutrophil functions remained intact, including neutrophil migration, phagocytosis, and release of important cytokines, such as IL-6, IL-1β, and TNF-α (22). In this study, we found that R406 was able to modulate the expression of most of the tested surface and intracellular features when NDNs and LDNs from patients with sepsis were stimulated with LPS. Moreover, this inhibition was also noted across both SYK^+^ and SYK^–^ subsets of NDNs and LDNs. These data demonstrate that SYK inhibition can regulate LPS activation of neutrophils from patients with sepsis, providing a mechanistic plausibility for how SYK inhibition may improve outcomes in sepsis. Last, SYK inhibition has been shown to inhibit the breakdown of VE-cadherin, decreasing endothelial cell permeability in the context of in vitro studies and a mouse model of sepsis-induced acute lung injury, providing additional preclinical data to support SYK inhibition in sepsis (42).

Emergency granulopoiesis including the release of immature neutrophils is an important part of sepsis pathogenesis (43). LDN-MC01 and LDN-MC02, which are immature MCs that express high levels of MPO, correlated with disease severity and expressed higher levels of SYK compared with LDN-MC03 and LDN-MC04. Furthermore, SYK^+^ WBNs and SYK^+^ LDNs expressed higher levels of CD33 consistent with immature neutrophils, while SYK^+^ LDNs expressed higher CD33 and lower CD10 than SYK^+^ WBNs. This data suggests that SYK expression may be a marker of neutrophil hematopoiesis. Furthermore, it suggests that mechanistically suppression of neutrophil hematopoiesis may explain prior COVID-19 data in which SYK inhibition led to stabilization in the proportion of circulating LDNs and immature neutrophils (17).

Our study has some limitations. First, this is a cross-sectional study, limiting our ability to describe trends in LDNs and SYK expression over time. Second, while there are clear differences in LDNs compared with WBNs, the WBN population contains some LDNs within it. Future studies to isolate NDNs and LDNs individually may provide further insight into the unique phenotypes of LDNs. Third, our ability to functionally characterize SYK^+^ and SYK^–^ neutrophil populations is limited to flow cytometry–based assays, as sorting based on intracellular features is not possible. Third, our analysis of SYK expression lacks evaluation in tissue compartments where neutrophils are more likely to contribute to pathogenesis. Last, prior in vivo work evaluating the efficacy of SYK inhibition in mouse models did not evaluate LDNs. Therefore, the applicability of our findings in the therapeutic setting of sepsis remains to be determined.

In conclusion, this study provides a comprehensive characterization of LDNs and SYK expression, providing a deeper understanding of signaling pathways involved in the pathogenesis of patients with sepsis. Specifically, we found that LDNs in sepsis are elevated, are phenotypically heterogenous including immature and hyperactivated subsets, and associate with the need for mechanical ventilation. Furthermore, SYK expression was increased in WBNs and LDNs, associated with disease severity along with many soluble biomarkers of immune activation, while SYK inhibition was able to inhibit the activation of neutrophils from patients with sepsis following LPS stimulation. Future studies to test the efficacy of SYK inhibition in large animal models may provide evidence to pursue SYK inhibition in human clinical trials of sepsis.

## Methods

### Sex as a biological variable.

Our study included both male and female patients with similar distributions. Findings are expected to be similar for both sexes.

### Patient enrollment.

Patients greater than 18 years old with sepsis diagnosed for less than 72 hours, defined using Sepsis-3 clinical criteria (suspected organ dysfunction and evidence of end-organ dysfunction [SOFA ≥ 2]) were enrolled at Inova Fairfax Hospital (IRB protocol 15-1863). Blood draw occurred the day after enrollment, and samples were transferred to the NIH Clinical Center for processing within 3 hours of blood draw. Age- and sex-matched HD blood samples were obtained from the NIH Department of Transfusion Medicine in Bethesda, Maryland, USA (IRB protocol 99-CC-0168) (Table 1).

### Flow cytometry analysis of WBNs.

Whole blood from patients with sepsis was obtained in EDTA tubes. Following 7-minute centrifugation at 1,400 rpm, plasma was aliquoted into cryovials. Erythrocytes were lysed with ACK Lysis Buffer (Quality Biological), and leukocytes were washed with fluorescence-activated cell sorting (FACS) buffer in conical tubes at room temperature. Next, 1 million leukocytes were aliquoted into FACS tubes on ice. Samples were blocked with FcR block for 10 minutes (Miltenyi Biotec) and stained with extracellular antibodies, pertaining to neutrophil maturation, degranulation, and activation (Supplemental Table 10). Leukocytes were then stained with Fixable Far-Red Dye (Life Technologies, Thermo Fisher Scientific) for viability. Subsequently, permeabilization was performed using Cytofix/Cytoperm (BD Biosciences). Intracellular antibodies were then added to each sample and washed. Samples were fixed in 1.6% paraformaldehyde and stored at 4°C before performing flow cytometry. Samples were acquired using a 4-laser Attune NxT flow cytometer (Thermo Fisher Scientific). WBNs were identified with traditional gating strategies. Following singlet gating, lymphocytes (CD3 and CD19), natural killer cells (CD56), thymocytes (CD2), and eosinophils (SIGLEC8) were removed in the channel designated as “DUMP,” which also includes live/dead (viability) staining. Additionally, neutrophil subsets were gated for CD15^+^ (Supplemental Figure 1, A and B). Fluorescence minus one (FMO) (Supplemental Figure 1C) samples were used to establish gating of samples. Additionally, SYK and MPO FMOs were acquired daily.

### Processing PBMCs containing LDNs.

Whole blood was mixed at a 1:1 ratio with phosphate-buffered saline (PBS) plus 2% fetal bovine serum (FBS) (StemCell Technologies) and layered onto Lymphoprep (StemCell Technologies) in filtered SepMate tubes (StemCell Technologies). PBMCs containing LDNs were spun at 1,200*g* for 15 minutes and washed with PBS plus 2% FBS. Red blood cells were lysed via ACK Lysis Buffer (Quality Biological), washed with PBS plus 2% FBS, and resuspended in FACS buffer. Subsequent staining and flow cytometry data acquisition followed the same method as described above for WBNs. The same traditional gating strategy used to identify WBNs was used for LDNs (Supplemental Figure 1B).

### Flow cytometry analysis.

Characterization of neutrophil subsets within WBNs and LDNs in sepsis and HD was previously described (21). Briefly, high dimensional flow cytometry data were analyzed using OMIQ (Dotmatics) software following a traditional gating strategy (Supplemental Figure 1) and a dimensional reduction approach. Dimensional reduction and clustering were completed on a subset of 23 patient samples to ensure adequate LDN counts and minimal shifts in the MPO signal. For dimensional reduction, we used optSNE on CD15^+^ WBNs or LDNs. FlowSOM algorithm was used for clustering. Two types of analyses were completed: (a) dimensional reduction and clustering within HD and sepsis WBNs and LDNs using 5,000 CD15^+^ events per FCS file; (b) dimensional reduction and clustering within LDNs using 5,000 CD15^+^ events per FCS file. Clustered heatmaps were generated in OMIQ according to applicable MC gating or using traditional flow cytometry gating to characterize SYK^+^ and SYK^–^ neutrophil populations.

### Measurement of soluble biomarkers.

Plasma levels of neutrophil-associated markers [MPO, neutrophil elastase (Elastase-2), lactoferrin, S100A8/MRP8, NGAL/LCN2, resistin], immunoregulatory cytokines [G-CSF, GM-CSF, IFN-γ, IL-1β, IL-6, IL-8, IL-10, IL-18, TNF-α, IL-1RA, TNF-RI, TNF-RII], and acute phase and endothelial activation markers [CRP, ICAM-1, MCP-1, MMP-9, SAA, VCAM-1] were measured using R-Plex and custom multiplexed U-Plex immunoassay [Meso Scale Discovery, MSD] following the manufacturer’s recommendation. Additionally, MPO levels were measured in the supernatants of LPS-stimulated NDNs and LDNs. The plates were coated with biotinylated antibodies combined with linkers and incubated overnight at 4°C. Standards and diluted samples were added to the plate in duplicate and incubated for 1–2 hours. After incubation time, plates were washed 3 times with PBS-Tween 0.05%. Then, the detection antibody solution was added, followed by 1-hour incubation. A final wash step was performed before adding Read Buffer. The plate was immediately read using the Meso QuickPlex SQ 120, and analyte concentrations were determined using Discovery Workbench 4.0 Analysis Software. Samples with undetectable biomarker levels were reported as a lower limit of detection for statistical purposes.

### Measurement of soluble NETs.

NET formation was measured using the MPO-DNA complex immunoassay. A clear, flat-bottom, immune, nonsterile, 96-well plate (Thermo Fisher Scientific 467340) was coated with 100 μL of anti-MPO diluted in PBS (1:1,000; Merck Millipore 07-496-I) and incubated at 4°C overnight. Following the overnight incubation period, plates were washed 5 times with PBS, and blocking was performed with 5% Albumin Bovine, Immunoassay Grade (Thermo Fisher Scientific J65731.30), at room temperature for 2 hours. Next, plates were washed 3 times with PBS. Subsequently, 100 μL of diluted plasma (1:4) was added in the wells and incubated overnight at 4°C, followed by 5 washes with 200 μL of PBS with 0.05% Tween. Then, 100 μL of anti-DNA antibody, double stranded (Sigma Aldrich MAB030), diluted 1:100 in 1% Albumin Bovine, was added to the wells and incubated at room temperature for 3 hours. After a 3-hour incubation, the plate was washed 5 times with 200 μL PBS with 0.05% Tween, and 100 μL of IgG HRP diluted 1:20,000 in 1% Albumin Bovine was added to the wells and incubated at room temperature for 1 hour. The plates were then washed with 200 μL of PBS-Tween with 0.05%, allowing each wash to stand for 5 minutes at room temperature. Then, 100 μL of 3,3’,5,5’-Tetramethylbenzidine (Life Technologies, Thermo Fisher Scientific; N301) was added in the wells and incubated in the dark for 25 minutes. The reaction was stopped by pipetting 100 μL of Stop solution (0.16 M sulfuric acid) (Life Technologies, Thermo Fisher Scientific; N600) per well, and absorbances were measured at 450 nm and 562 nm (background) using CLARIOstar Plus microplate reader (BMG LabTech). The results are expressed as optical density, at a wavelength of 450 nm and subtracting the 562 nm.

### Stimulation of neutrophils with LPS.

NDNs and LDNs were isolated from EDTA whole blood from patients with sepsis (44). In detail, total neutrophils were isolated from 10 mL of EDTA whole blood using the StemCell EasySep Direct Human Neutrophils Isolation Kit (19666, StemCell Technologies). Total neutrophils were centrifuged and resuspended in 2 mL of Dulbecco’s Phosphate Buffered Saline with 2% FBS (07905, StemCell Technologies) and layered on 4 mL of Lymphoprep (07811, StemCell Technologies). Following centrifugation, the LDNs were collected from above the Lymphoprep and NDNs from below the Lymphoprep into a separate tube containing 10 mL Dulbecco’s PBS + 2% FBS and centrifuged at 300*g* for 5 minutes. Following centrifugation, NDNs and LDNs were collected in X-VIVO-15 serum-free medium (Lonza Bioscience).

Purified NDNs or LDNs were preincubated for 30 minutes with 2 μM R406 (provided by Rigel Pharmaceuticals) or media and stimulated with 1 μg/mL of LPS from *Klebsiella pneumoniae* (MilliporeSigma, L4268) at 37°C for 1 hour. The supernatants were collected to measure MPO by MSD, and NDNs and LDNs were stained for flow cytometry (Supplemental Table 10).

### Statistics.

Flow cytometry data from OMIQ Software were exported into Microsoft Excel and GraphPad Prism (version 10.4.1) for downstream analyses. The D’Agostino normality test determined if data followed a normal Gaussian distribution. Statistical significance for 2 groups was measured by 2-tailed Mann-Whitney nonparametric *t* test or a parametric *t* test if unpaired. However, if groups were being compared within a sample group (i.e., sepsis WBNs versus sepsis LDNs), significance was measured by 2-tailed Wilcoxon’s paired nonparametric test or a parametric paired *t* test. For multiple-group comparisons, significance was determined using a 1-way ANOVA with a Tukey’s multiple comparisons test, a Kruskal-Wallis with a Dunn multiple-comparison test, or a Conover post hoc test for Friedman’s test with a Bonferroni correction. For categorical variables, significance was determined by a Fisher’s exact test when comparing 2 groups or a Pearson’s χ^2^ test when comparing multiple groups. Correlation coefficients were calculated using Pearson’s correlation coefficient or Spearman’s rank correlation coefficient test for continuous variables and a point-biserial correlation between dichotomous and continuous variables. All data are shown as mean ± SEM. *P* values ≤ 0.05 were considered statistically significant. Random Forest classification was implemented in R version 4.4.3 (R Core Team, 2024) using the randomForest package version 4.7-1.2. The dependent variable was mechanical ventilation status (binary), and predictors included age, sex, WBC count, 24 soluble biomarkers, 22 whole-blood neutrophil biomarkers, and 22 LDN biomarkers.

### Study approval.

This study was approved by the WCG IRB at INOVA Fairfax Hospital (IRB15-1863), and written informed consent was obtained from all patients or a legally authorized representative.

### Data availability.

Values for all data points found in graphs are in the Supporting Data Values file.

## Author contributions

JRS acquired funding and supervised the project. Study conceptualization was completed by JRS and HLT. Experimental methodologies were designed and optimized by JRS, HLT, and XY. The clinical cohort was established and consented and samples were collected by JRS, CSK, SDN, JDW, AH, EE, and MW. Sample processing and data collection were completed by LK, HLT, RSDC, XY, and KA. Data analysis and presentation were completed by HLT, LK, RSDC, XY, TW, and CD.

## Conflict of interest

The authors have declared that no conflict of interest exists.

## Funding support

This work is the result of NIH funding, in whole or in part, and is subject to the NIH Public Access Policy. Through acceptance of this federal funding, the NIH has been given a right to make the work publicly available in PubMed Central.

- Intramural Research Program of the NIH.

## Supplementary Material

Supplemental data

Supporting data values

## Figures and Tables

**Table 1:**
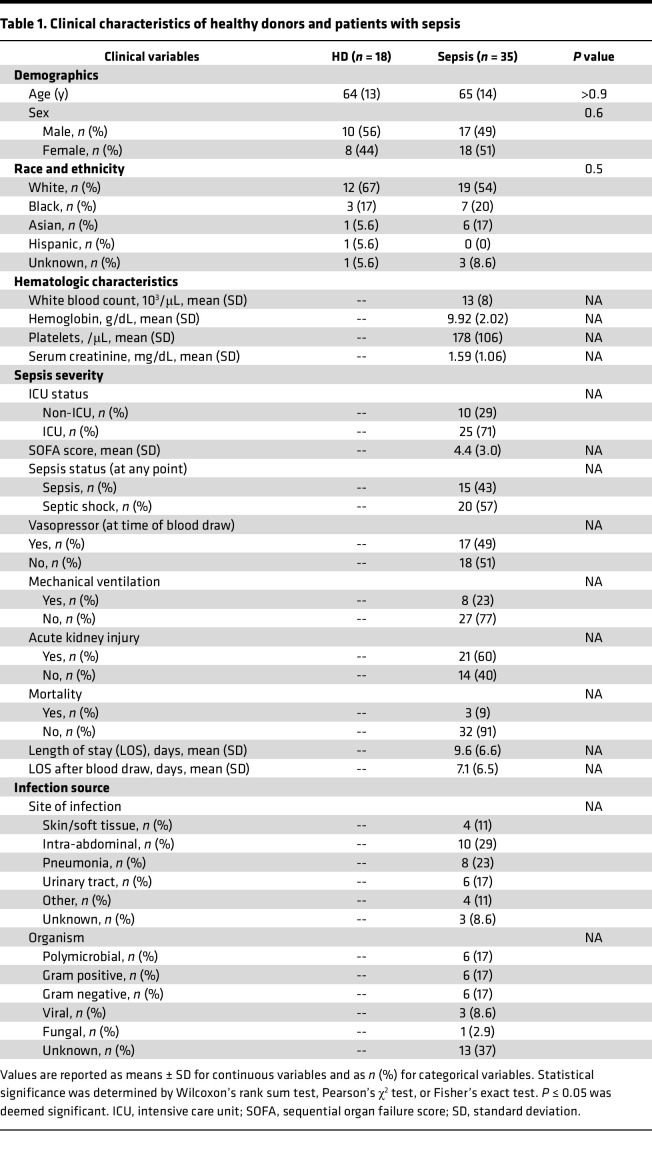
Clinical characteristics of healthy donors and patients with sepsis

## Version 1 04/21/2026

In-Press Preview

## Version 2 06/08/2026

Electronic publication
