# Supplementary material for: Low-density neutrophil heterogeneity and spleen tyrosine kinase as therapeutic targets in sepsis
Source: JCI Insight. 2026 Apr 21;11(11):e201057. doi: 10.1172/jci.insight.201057 (PMC13313530; doi:10.1172/jci.insight.201057)
Supplement: Supplemental data [file jciinsight-11-201057-s155.pdf]

Supplemental Material

Low Density Neutrophil Heterogeneity and Spleen Tyrosine Kinase as a Therapeutic Target in Sepsis

Heather L. Teague[1,2], Lauren Knabe[1,2], Raquel Cruz[1,2], Xianglan Yao[1,2], Kiana Allen[1,2], Trenton Williams[1,2], Cumhur Y. Demirkale[2], Merte Lemma Woldehanna[3], Ernest Evans[3], Amir Hobson[3], Jared Wilkinson[3], Steven D. Nathan[3], Christopher King[3], Jeffrey R. Strich[1,2*]

1. Critical Care Medicine and Pulmonary Branch, National Heart Lung and Blood Institute, National Institutes of Health, Bethesda, MD, USA
2. Critical Care Medicine Department, Clinical Center, National Institutes of Health, Bethesda, MD, USA
3. Advanced Lung Disease and Lung Transplant Program, Inova Fairfax Hospital, Falls Church, VA, US

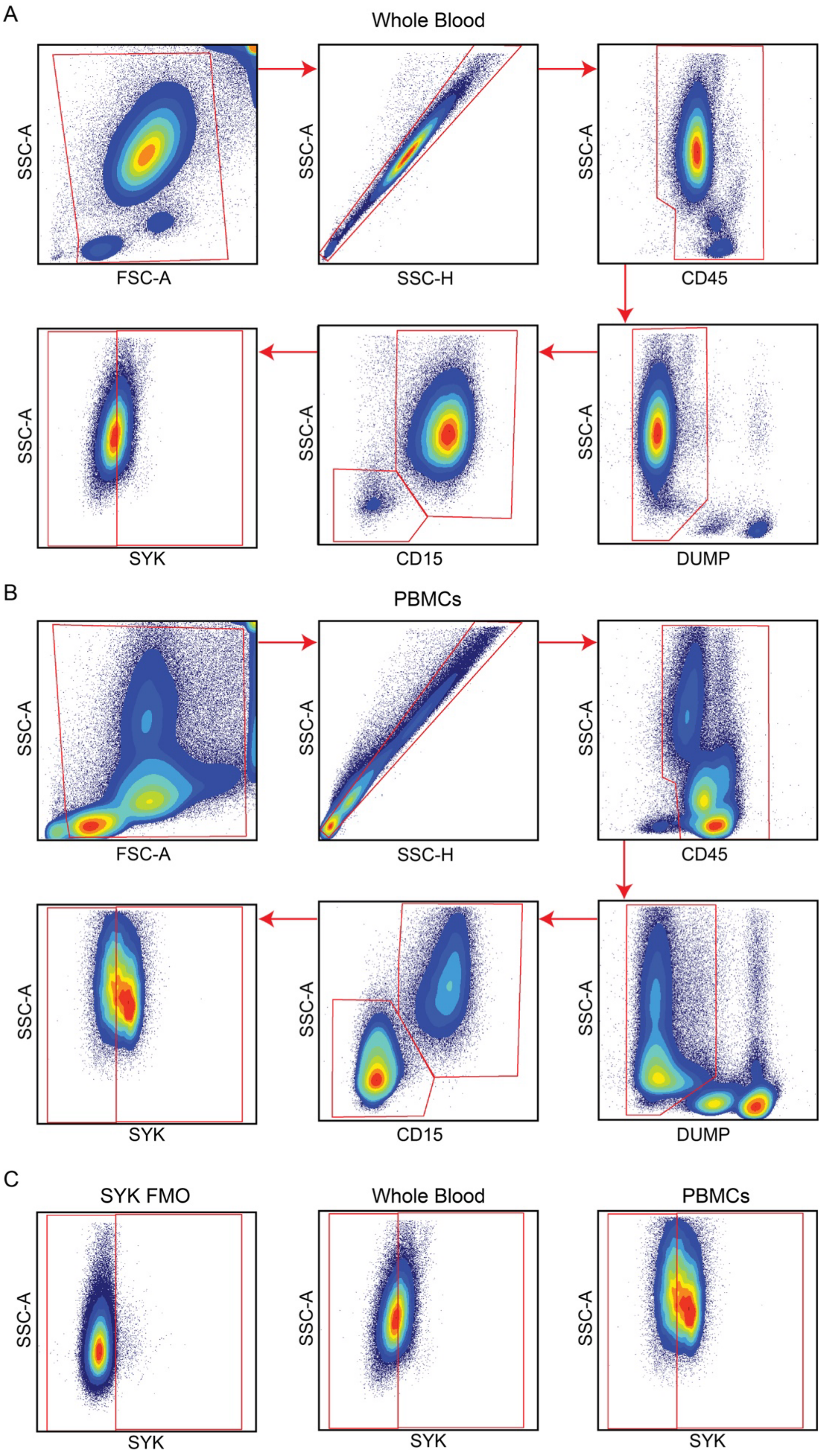
A
Whole Blood
SSC-A
FSC-A
SSC-H
CD45
SYK
CD15
DUMP
B
PBMCs
C
SYK FMO
Whole Blood
PBMCs

Supplemental Figure 1. Neutrophil traditional flow cytometry gating strategy. (**A**) Gating strategy to identify $CD15^+$ whole blood neutrophils (WBNs). (**B**) Gating strategy to identify $CD15^+$ low-density neutrophils (LDNs) within the peripheral blood mononuclear fraction (**C**). Representative SYK FMO gating strategy for whole blood and PBMCs samples. PBMCs: peripheral blood mononuclear cells; SSC: side scatter; FSC: forward scatter; A: area; H: height; DUMP: channel staining for B Cells, T Cells, and NK cells for removal; SYK: spleen tyrosine kinase, FMO:fluorescence minus one.

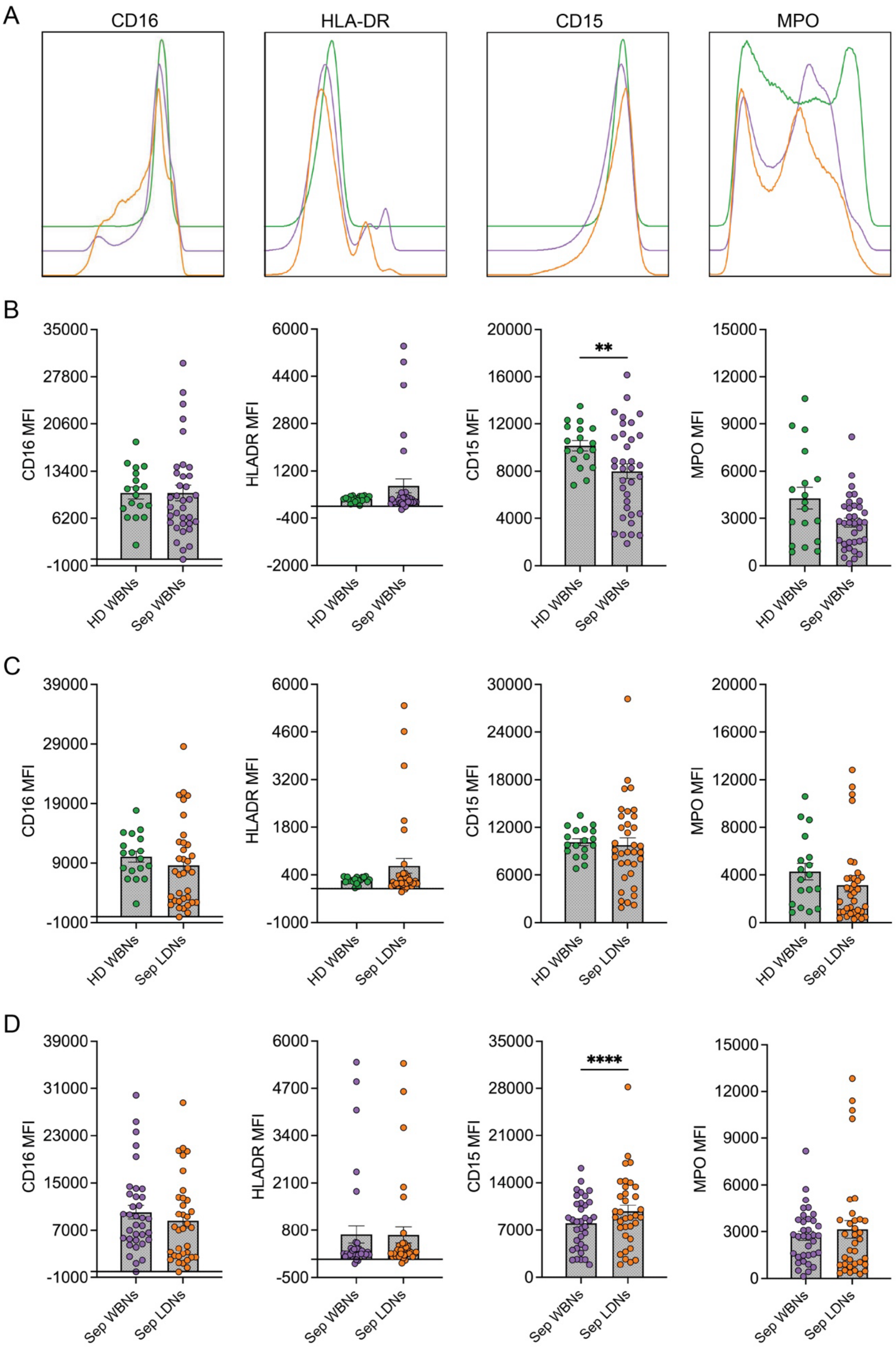
A
CD16
HLA-DR
CD15
MPO
B
CD16 MFI
HLADR MFI
CD15 MFI
MPO MFI
**
HD WBNs
Sep WBNs
C
HD WBNs
Sep LDNs
D
****
Sep WBNs
Sep LDNs

Supplemental Figure 2. Characterization of sepsis WBNs and LDNs and healthy donor WBNs. (**A**) Histogram demonstrating the relative expression, MFI, of features between healthy donor WBNs and sepsis WBNs and LDNs. (**B**) Bar graphs comparing the relative expression of features between healthy donor and sepsis WBNs. (**C**) Bar graphs comparing the relative expression of features between healthy donor WBNs and sepsis LDNs. (**D**) Bar graphs comparing the relative expression of features between sepsis WBNs and LDNs. Data are represented as means±SEM. Significance was determined using a Mann-Whitney test, Paired t-test or a Wilcoxon test and set at *P*<0.01** and *P*<0.0001****. WBNs: whole blood neutrophils; LDNs: low-density neutrophils; MFI: mean fluorescence intensity; MPO: myeloperoxidase; SEM: standard error of the mean.

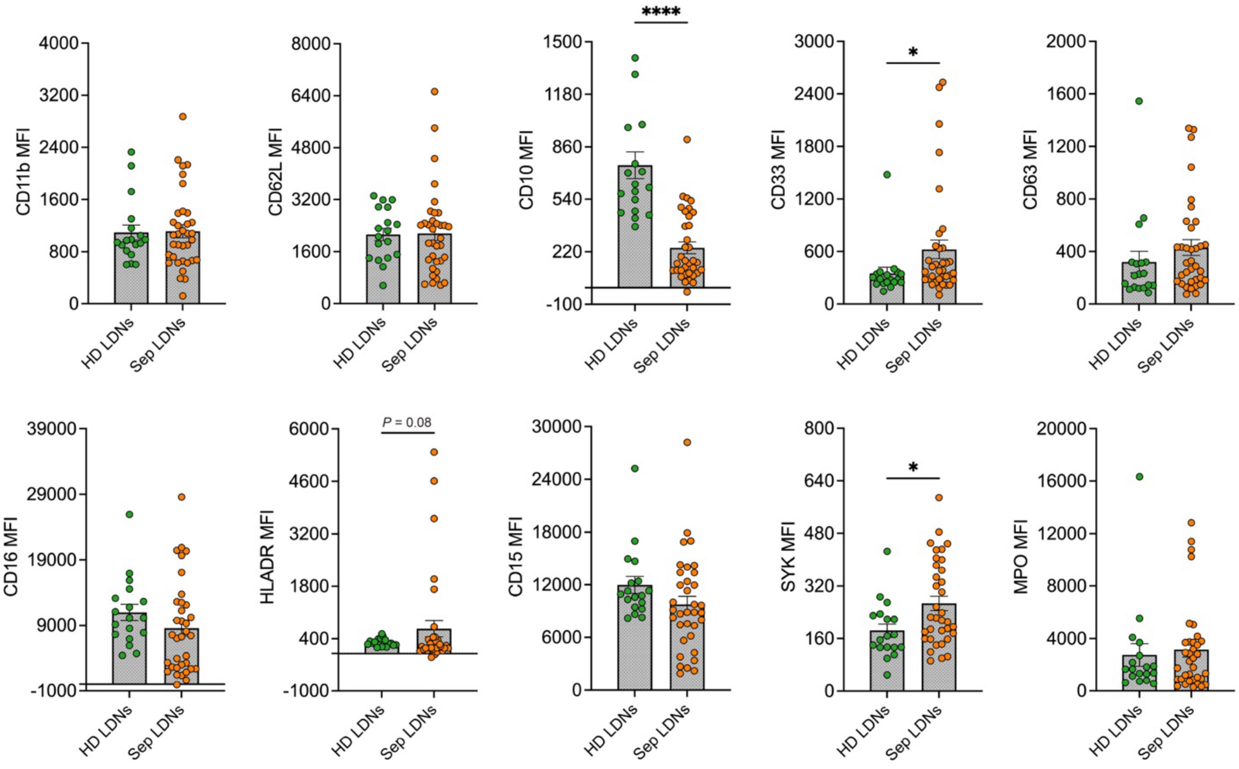

Supplemental Figure 3. Characterization of healthy donor and sepsis LDNs. Bar graphs comparing the relative expression of features between healthy donor and sepsis LDNs. Data are represented as means±SEM. Significance was determined using a Mann-Whitney test and set at $P<0.05$* and $P<0.0001$****. WBNs: whole blood neutrophils; LDNs: low-density neutrophils; MFI: mean fluorescence intensity; SEM: standard error of the mean.

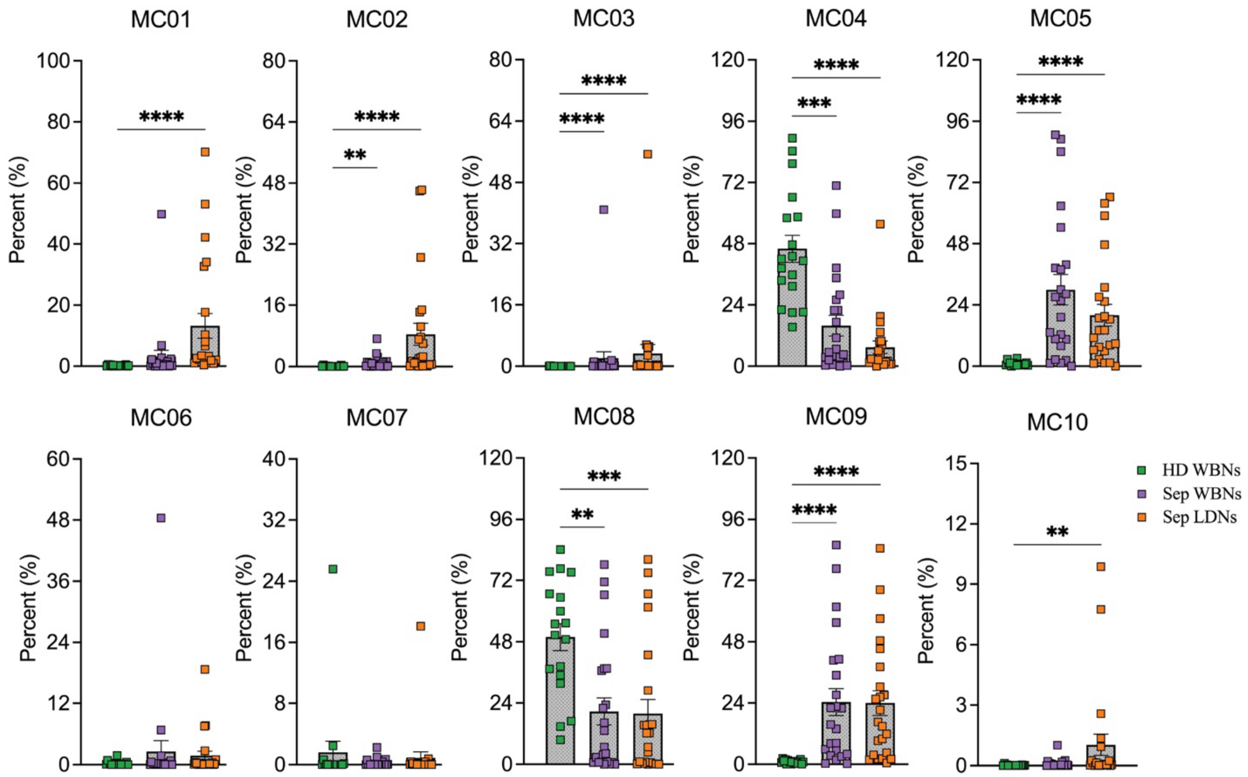

Supplemental Figure 4. Metacluster percent across each neutrophil group. Within the healthy donor and sepsis neutrophil samples, 10 metaclusters were identified with varying percentages per metacluster. Data are represented as means±SEM. Significance was determined using a Kruskal-Wallis with a Dunn's multiple comparison test and set at P<0.01**, P<0.001***, P<0.0001****. HD: healthy donor; Sep: sepsis; WBNs: whole blood neutrophils; LDNs: low-density neutrophils; MFI: mean fluorescence intensity; MC: metacluster; SEM: standard error of the mean.

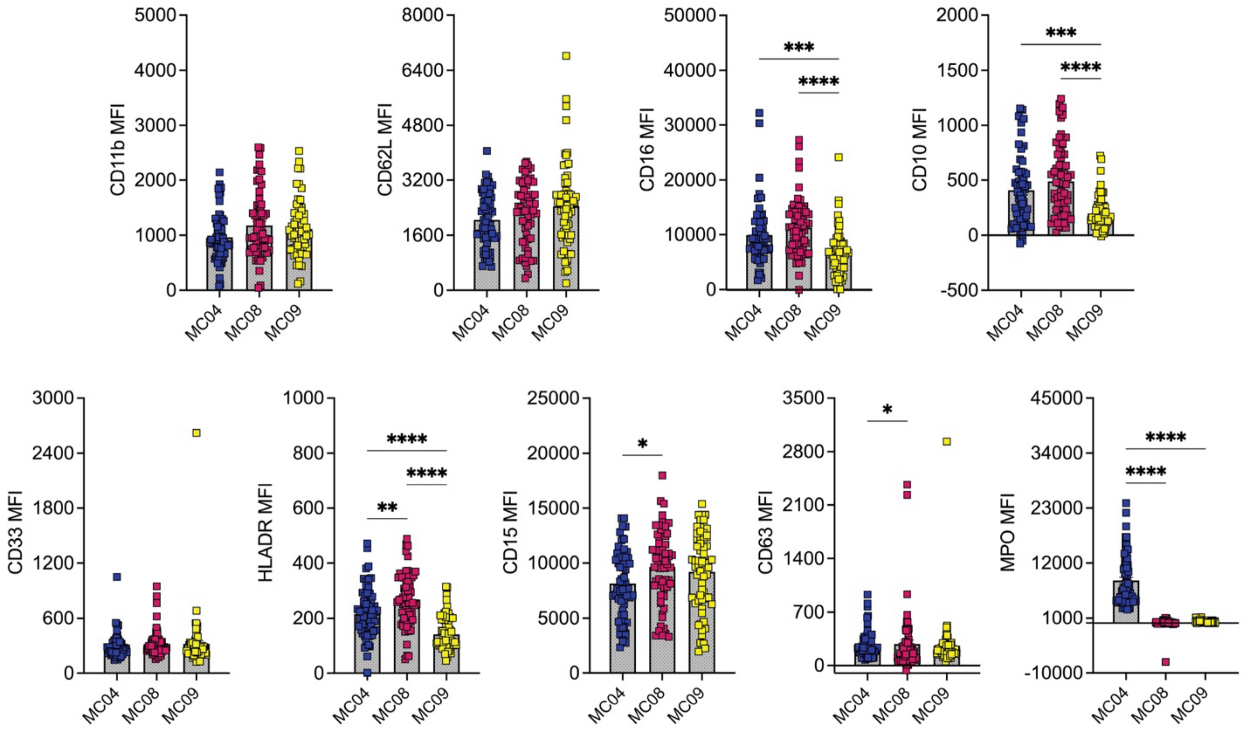

Supplemental Figure 5. Comparison of features describing metaclusters that are prominent in healthy donors compared to sepsis WBNs and LDNs. Characterization of the expression level of each feature comparing MC04, MC08, and MC09. Data are represented as means±SEM. Significance was determined using a One-way ANOVA with a Tukey's Multiple Comparisons test or a Kruskal-Wallis with a Dunn's multiple comparison test and set at $P<0.05$*, $P<0.01$**, $P<0.001$***, $P<0.0001$****. MC: metacluster; MFI: mean fluorescence intensity; SEM: standard error of the mean.

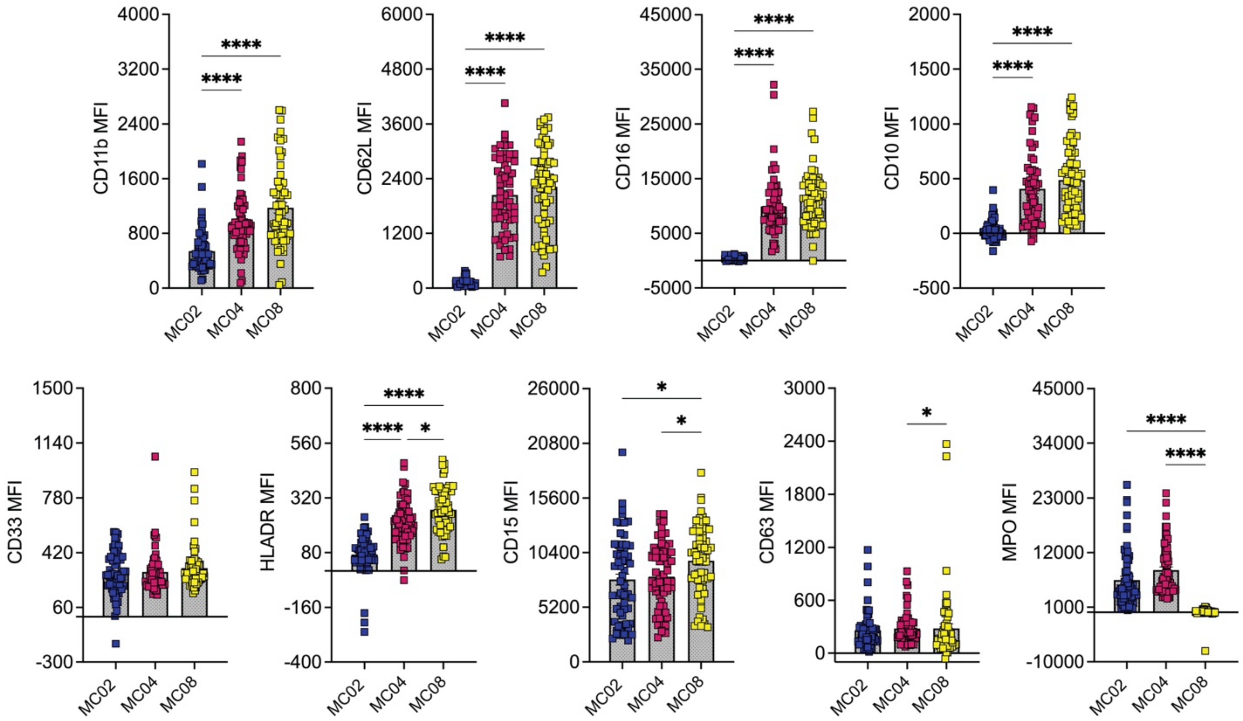

Supplemental Figure 6. Comparison of features describing metaclusters that are prominent in healthy donors compared to sepsis LDNs. Characterization of the expression level of each feature comparing MC02, MC04, and MC08. Data are represented as means±SEM. Significance was determined using a One-way ANOVA with a Tukey's Multiple Comparisons test or a Kruskal-Wallis with a Dunn's multiple comparison test and set at *P*<0.05*, *P*<0.0001****. MC: metacluster; MFI: mean fluorescence intensity; SEM: standard error of the mean.

A

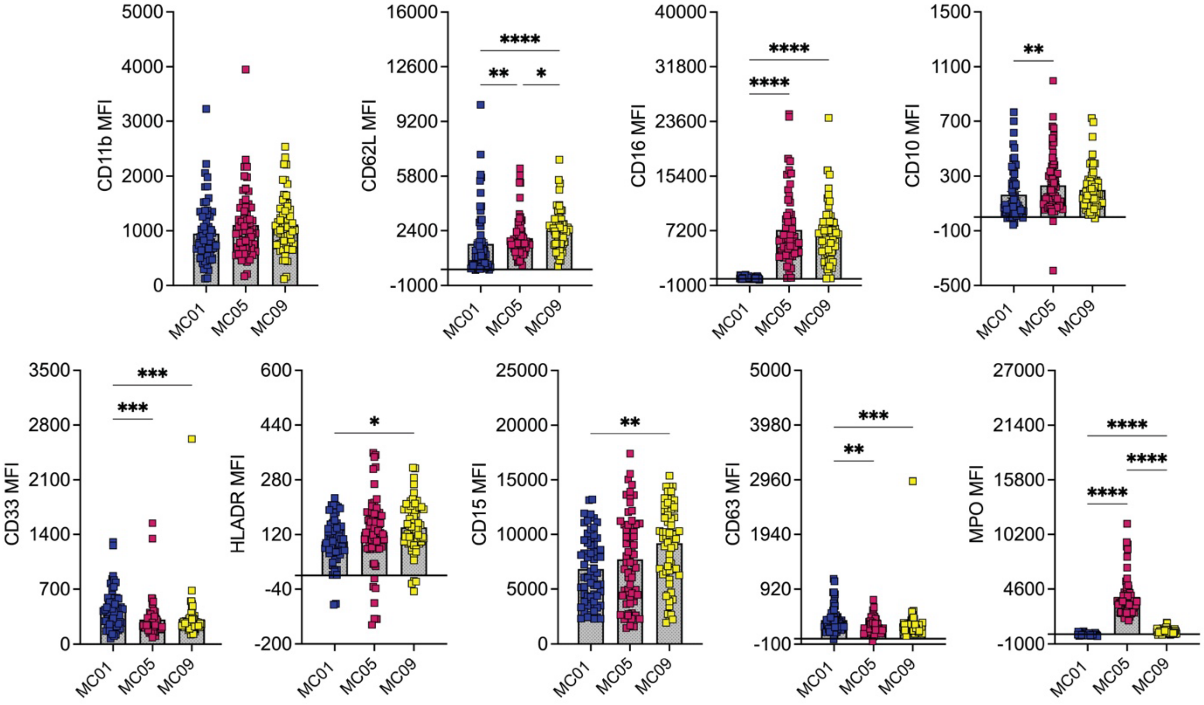

B

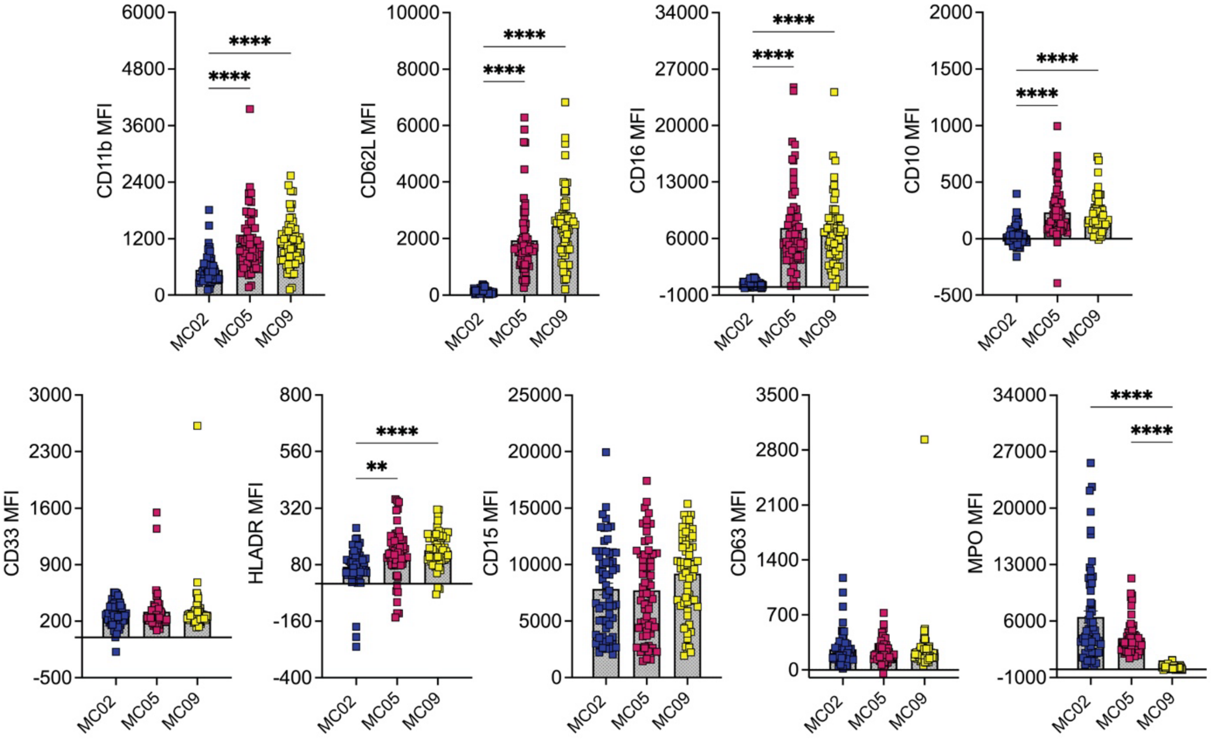

Supplemental Figure 7. Comparison of features describing metaclusters that are prominent in sepsis WBNs and LDNs. Characterization of the expression level of each

feature comparing (**A**) MC01, MC05, and MC09 and (**B**) MC02, MC05, and MC09. Data are represented as means±SEM. Significance was determined using a One-way ANOVA with a Tukey's Multiple Comparisons test or a Kruskal-Wallis with a Dunn's multiple comparison test and set at $P<0.05$*, $P<0.01$**, $P<0.001$***, $P<0.0001$****. MC: metacluster; MFI: mean fluorescence intensity; SEM: standard error of the mean.

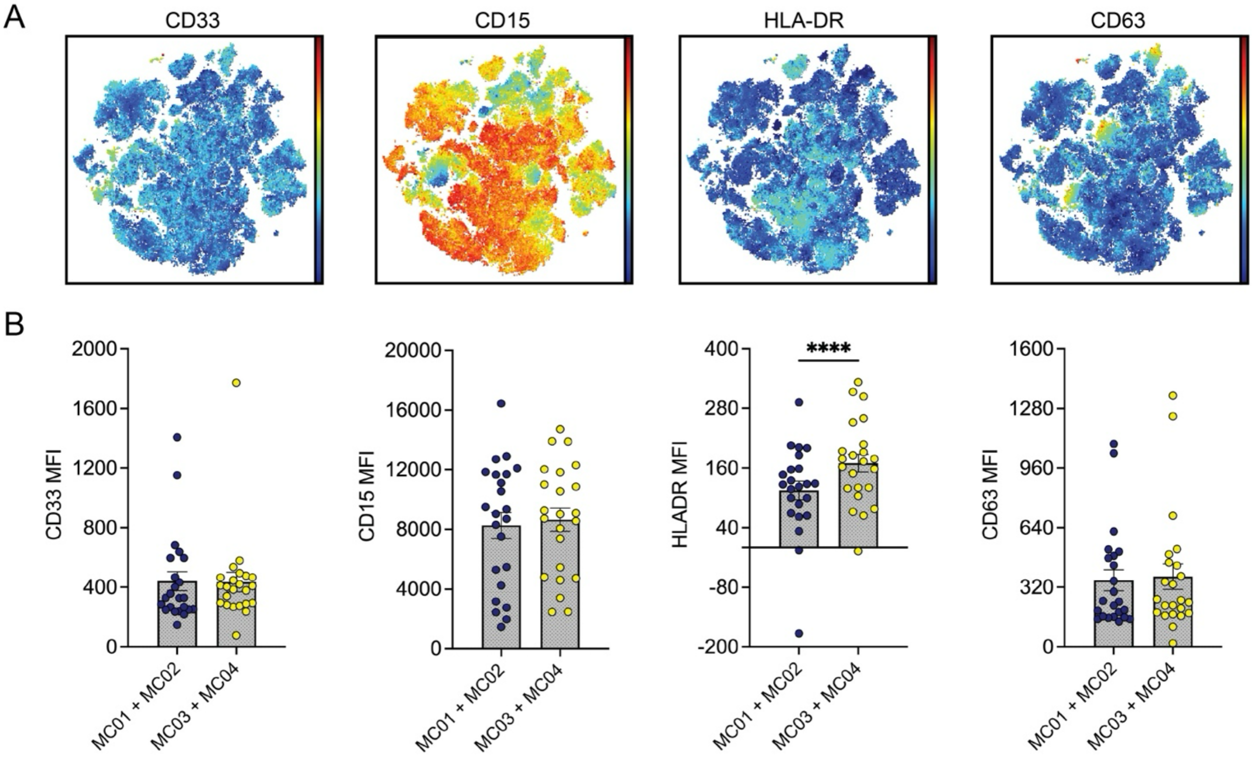

Supplemental Figure 8. Features of sepsis LDNs. Characterization of the metaclusters identified within sepsis LDNs. (**A**) optSNE plots of LDN features. (**B**) Bar graphs representing the expression of features in LDN-MC01 and LDN-MC02 compared to LDN-MC03 and LDN-MC04. Data are represented as means±SEM. Significance was determined using a Paired t-test or a Wilcoxon test and set at *P*<0.0001****. MC: metacluster; MFI: mean fluorescence intensity; SEM: standard error of the mean.

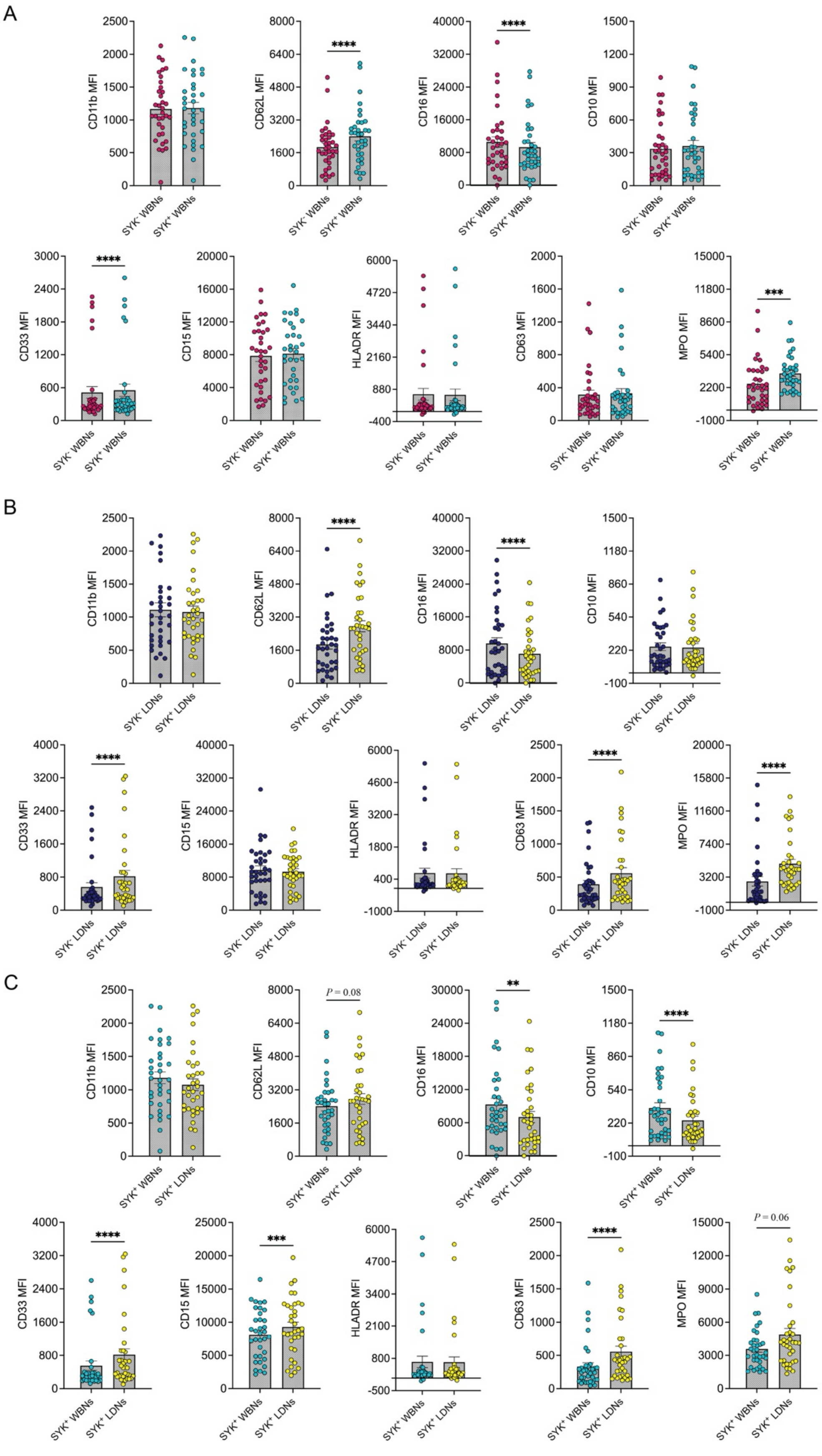
A
CD11b MFI
CD62L MFI
CD16 MFI
CD10 MFI
CD33 MFI
CD15 MFI
HLADR MFI
CD63 MFI
MPO MFI
SYK⁻ WBNs
SYK⁺ WBNs
****
***
B
SYK⁻ LDNs
SYK⁺ LDNs
C
SYK⁺ WBNs
SYK⁺ LDNs
P = 0.08
P = 0.06
**

Supplemental Figure 9. Characterization of SYK$^+$ versus SYK$^-$ neutrophil populations. Comparison of features between SYK$^+$ and SYK$^-$ (**A**) WBNs, (**B**) LDNs and (**C**) SYK$^+$WBNs and SYK$^+$LDNs. Data are represented as means±SEM. Significance was determined using a Paired t-test or a Wilcoxon test and set at *P*<0.01**, *P*<0.001***, *P*<0.0001****. WBNs: whole blood neutrophils; LDNs: low-density neutrophils; SYK: spleen tyrosine kinase; MFI: mean fluorescence intensity; SEM: standard error of the mean.

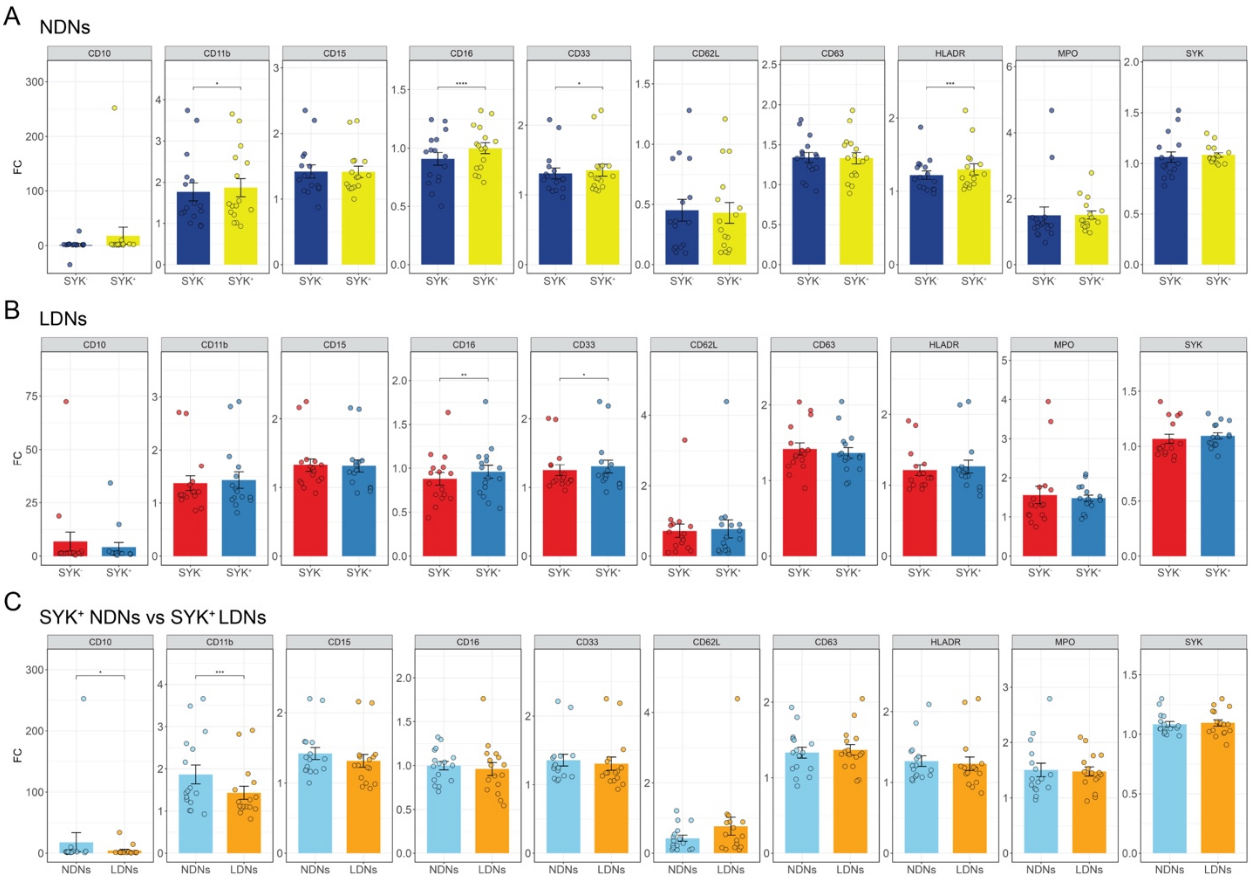

Supplemental Figure 10. Functional assessment of SYK$^+$ and SYK$^-$ neutrophils following LPS stimulation and SYK inhibition. Bar graphs representing the fold change in feature expression following LPS stimulation comparing NDNs and LDNs from (**A**) SYK$^-$ vs SYK$^+$ NDNs, (**B**) SYK$^-$ vs SYK$^+$ LDNs, (**C**) SYK$^+$ NDNs vs SYK$^+$ LDNs. Significance was determined using a Wilcoxon test and set at *P*<0.05*, *P*<0.01**, *P*<0.001***, *P*<0.001***. MFI: mean fluorescence intensity; NDNs: normal density neutrophils; LDNs: low-density neutrophils; LPS: lipopolysaccharide; SEM: standard error of the mean; FC: fold change.

Supplemental Table 1. Clinical Characteristics of 23 patients from high dimensional flow cytometry analysis

Clinical Variables	Sepsis Dimensional Reduction (*n* = 23)	Sepsis Entire Cohort (*n* = 35)	*P*-value
**Demographics**			
Age (years)	63 (15)	65 (14)	0.7
Sex			0.7
Male, *n (%)*	10 (43)	17 (49)	
Female, *n (%)*	13 (57)	18 (51)	
Race			0.9
White	10 (43)	19 (54)	
Black	5 (22)	7 (20)	
Asian	6 (26)	6 (17)	
Unknown	2 (8.7)	3 (8.6)	
**Hematologic Characteristics**			
White Blood Count, 10^3/uL, *mean (SD)*	15 (10)	13 (8)	0.4
Hemoglobin, g/dL, *mean (SD)*	10.14 (2.14)	9.92 (2.02)	0.7
Platelets, /uL, *mean (SD)*	195 (118)	178 (106)	0.6
Serum Creatinine, mg/dL, *mean (SD)*	1.48 (0.90)	1.59 (1.06)	0.8
**Sepsis Severity**			
ICU Status			0.6
Non-ICU, *n (%)*	5 (22)	10 (29)	
ICU, *n (%)*	18 (78)	25 (71)	
SOFA Score, *mean (SD)*	4.4 (3.2)	4.4 (3.0)	>0.9
Sepsis Status (at any point)			0.8
Sepsis, *n (%)*	9 (39)	15 (43)	
Septic Shock, *n (%)*	14 (61)	20 (57)	
Vasopressor (at time of blood draw)			0.8
Yes, *n (%)*	22 (48)	17 (49)	
No, *n (%)*	11 (52)	18 (51)	
Mechanical Ventilation			0.5
Yes, *n (%)*	7 (30)	8 (23)	
No, *n (%)*	16 (70)	27 (77)	
Acute Kidney Injury			0.8
Yes, *n (%)*	13 (57)	21 (60)	
No, *n (%)*	10 (43)	14 (40)	
Mortality			>0.9
Yes, *n (%)*	2 (9)	3 (9)	
No, *n (%)*	21 (91)	32 (91)	
Length of Stay (LOS), days, *mean (SD)*	10.2 (7.1)	9.6 (6.6)	0.7

LOS After Blood Draw, days, *mean (SD)*	8 (7)	7.1 (6.5)	0.6
**Infection Source**			
Site of Infection			>0.9
Skin/soft tissue, *n (%)*	3 (13)	4 (11)	
Intra-abdominal, *n (%)*	5 (22)	10 (29)	
Pneumonia, *n (%)*	6 (26)	8 (23)	
Urinary Tract, *n (%)*	5 (22)	6 (17)	
Other, *n (%)*	3 (13)	4 (11)	
Unknown, *n (%)*	1 (4)	3 (8.6)	
Organism			>0.9
Polymicrobial, *n (%)*	5 (22)	6 (17)	
Gram Positive, *n (%)*	4 (17)	6 (17)	
Gram Negative, *n (%)*	4 (17)	6 (17)	
Viral, *n (%)*	2 (8.7)	3 (8.6)	
Fungal, *n (%)*	1 (4.3)	1 (2.9)	
Unknown, *n (%)*	7 (30)	13 (37)	

Abbreviations: ICU: Intensive care unit; SOFA: Sequential organ failure score; SD: Standard deviation.

Supplemental Table 2. Abundance summary of 10 metaclusters comparing healthy donor whole blood neutrophils to sepsis WBNs and LDNs

	**HD WBNs (*n* = 18)**	**Sepsis WBNs (*n* = 23)**	**Sepsis LDNs (*n* = 23)**	***P*-value**
MC01, *mean (SD)*	0 (0)	3 (10)	13 (19)	<0.0001
MC02, *mean (SD)*	0.1 (0.1)	1.0 (1.6)	8.4 (13.7)	<0.0001
MC03, *mean (SD)*	0.02 (0.04)	2.07 (8.48)	3.34 (11.49)	<0.0001
MC04, *mean (SD)*	46 (22)	16 (20)	7 (12)	<0.0001
MC05, *mean (SD)*	1 (1)	30 (29)	20 (21)	<0.0001
MC06, *mean (SD)*	0.27 (0.44)	2.58 (10.09)	1.76 (4.27)	0.13
MC07, *mean (SD)*	1.62 (6.01)	0.24 (0.51)	0.90 (3.75)	0.79
MC08, *mean (SD)*	50 (23)	21 (25)	20 (26)	0.0006
MC09, *mean (SD)*	1 (1)	24 (25)	24 (23)	<0.0001
MC10, *mean (SD)*	0.02 (0.03)	0.09 (0.22)	1.03 (2.55)	0.009

Abbreviations: WBNs: whole blood neutrophils; LDNs: low density neutrophils; SD: standard deviation; MC: metacluster.

Supplemental Table 3. Corresponding values to volcano plot comparing healthy donor WBNs and sepsis WBNs

	**logFC**	***P*-value**	**FDR**	**log10(FDR)**
MC05	5.03	2.70E-15	2.70E-14	13.57
MC09	4.69	8.02E-14	4.01E-13	12.40
MC03	6.52	4.97E-08	1.66E-07	6.78
MC02	3.34	8.59E-07	2.15E-06	5.67
MC01	3.89	1.69E-06	3.39E-06	5.47
MC06	3.26	0.003	0.005	2.30
MC04	-1.54	0.005	0.006	2.22
MC07	-2.74	0.007	0.009	2.05
MC10	2.09	0.01	0.011	1.96
MC08	-1.27	0.03	0.03	1.47

Abbreviations: logFC: log fold change; FDR: false discovery rate; whole blood neutrophils: WBNs.

Supplemental Table 4. Corresponding values to volcano plot comparing healthy donor WBNs and sepsis LDNs

	**logFC**	***P*-value**	**FDR**	**log10 (FDR)**
MC01	5.98	2.02E-16	2.02E-15	14.69
MC09	4.66	6.00E-15	3.00E-14	13.52
MC02	6.43	1.24E-13	4.13E-13	12.38
MC05	4.44	2.08E-13	5.20E-13	12.28
MC03	7.21	4.45E-08	8.89E-08	7.05
MC10	5.53	2.05E-07	3.42E-07	6.47
MC04	-2.63	3.78E-07	5.39E-07	6.27
MC06	2.71	0.001	0.001	3.00
MC08	-1.33	0.03	0.034	1.47
MC07	-0.85	0.417	0.417	0.38

Abbreviations: logFC: log fold change; FDR: false discovery rate; MC: metacluster; WBNs: whole blood neutrophils; LDNs: low density neutrophils.

Supplemental Table 5. Corresponding values to volcano plot comparing sepsis WBNs to sepsis LDNs

	**logFC**	***P*-value**	**FDR**	**log10(FDR)**
MC02	3.09	5.43E-06	5.43E-05	4.27
MC10	3.44	0.0002	0.001	3.00
MC01	2.08	0.0031	0.0104	1.98
MC07	-1.89	0.0425	0.1062	0.97
MC04	-1.10	0.0754	0.1509	0.82
MC05	-0.59	0.2856	0.476	0.32
MC03	0.69	0.4351	0.6215	0.21
MC06	-0.55	0.5575	0.6969	0.16
MC08	-0.06	0.9335	0.9672	0.01
MC09	-0.02	0.9672	0.9672	0.01

Abbreviations: logFC: log fold change; FDR: false discovery rate; MC: metacluster; WBNs: whole blood neutrophils; LDNs: low density neutrophils.

Supplemental Table 6. Correlation of WBNs, LDNs, SYK^{+}WBNs and SYK^{+}LDNs with soluble biomarkers

	WBNs/CD45		LDNs/CD45		SYK^{+}WBNs/CD45		SYK^{+}LDNs/CD45	
**Biomarkers**	***R*-value**	***P*-value**	***R*-value**	***P* -value**	***R*-value**	***P* -value**	***R*-value**	***P* -value**
**Neutrophil-Associated Markers**								
Elastase-2	0.530	0.001	0.556	0.001	0.005	0.979	0.497	0.003
Lactoferrin	0.482	0.004	0.318	0.067	-0.077	0.665	0.222	0.206
MPO	0.148	0.403	0.142	0.425	0.030	0.866	0.127	0.473
MPO-DNA	0.041	0.816	0.096	0.589	0.076	0.671	0.089	0.616
NGAL	0.149	0.401	0.016	0.928	-0.193	0.275	-0.049	0.782
Resistin	0.161	0.364	0.289	0.097	-0.013	0.941	0.255	0.145
S100A8	0.441	0.009	0.393	0.022	0.047	0.792	0.402	0.019
**Inflammatory Markers**								
G-CSF	0.216	0.219	0.351	0.042	-0.058	0.745	0.306	0.079
IL-1β	0.142	0.422	0.537	0.001	-0.268	0.126	0.262	0.134
IL-1RA	0.275	0.116	0.452	0.007	-0.184	0.298	0.313	0.071
IL-6	0.173	0.327	0.005	0.977	0.051	0.772	0.111	0.532
IL-8	0.125	0.482	0.534	0.001	-0.076	0.670	0.417	0.014
IL-10	0.442	0.009	0.371	0.031	0.041	0.818	0.344	0.047
IL-18	0.104	0.558	0.320	0.065	0.258	0.140	0.427	0.012
IFN-γ	-0.121	0.496	-0.018	0.918	-0.246	0.161	-0.021	0.908
TNF-α	0.132	0.458	0.177	0.317	-0.115	0.517	0.066	0.711
TNF-RI	0.059	0.738	0.097	0.585	-0.171	0.334	-0.019	0.916
TNF-RII	0.688	<0.001	0.463	0.006	0.155	0.382	0.455	0.007
CRP	0.233	0.185	0.216	0.220	0.146	0.409	0.191	0.280
MCP-1	0.145	0.414	0.140	0.430	-0.200	0.257	0.050	0.779
SAA	0.375	0.029	0.236	0.179	0.280	0.109	0.240	0.172
**Endothelial Markers**								
ICAM-1	0.075	0.675	0.264	0.132	0.004	0.984	0.265	0.130
MMP-9	0.098	0.581	0.018	0.921	-0.133	0.454	-0.093	0.600
VCAM-1	-0.076	0.671	-0.024	0.895	-0.179	0.311	-0.178	0.315

Abbreviations: WBNs: whole blood neutrophils; LDNs: low density neutrophils; SYK: spleen tyrosine kinase. All data in the table are expressed as Spearman's correlation coefficient. Two-tailed $P \leq 0.05$ are deemed significant (bolded values).

Supplemental Table 7. Correlation of SYK⁺WBNs and SYK⁺LDNs by median levels with soluble biomarkers

	SYK⁺WBNs/CD45				SYK⁺LDNs/CD45			
	Below		Above		Below		Above	
**Biomarkers**	***R*-value**	***P*-value**	***R*-value**	***P* -value**	***R*-value**	***P* -value**	***R*-value**	***P* -value**
**Neutrophil-Associated Markers**								
Elastase-2	0.243	0.347	-0.061	0.816	0.400	0.113	0.654	0.005
Lactoferrin	0.056	0.832	-0.184	0.480	0.189	0.467	0.679	0.004
MPO	0.130	0.619	-0.154	0.553	0.062	0.814	0.520	0.035
MPO-DNA	0.057	0.829	0.063	0.811	-0.271	0.293	0.064	0.809
NGAL	-0.034	0.898	-0.049	0.854	0.025	0.928	0.547	0.025
Resistin	0.228	0.377	-0.319	0.212	-0.012	0.966	0.789	0.000
S100A8	0.253	0.372	0.140	0.592	0.277	0.281	0.615	0.010
**Inflammatory Markers**								
G-CSF	0.321	0.209	-0.291	0.257	0.309	0.227	0.424	0.091
IL-1β	0.311	0.224	-0.199	0.444	0.091	0.730	0.628	0.008
IL-1RA	0.064	0.809	-0.130	0.619	0.125	0.632	0.588	0.015
IL-6	0.287	0.264	-0.294	0.251	-0.140	0.592	0.539	0.028
IL-8	0.311	0.224	-0.128	0.625	-0.012	0.966	0.650	0.006
IL-10	0.380	0.133	0.049	0.853	0.485	0.050	0.574	0.018
IL-18	0.338	0.184	-0.355	0.162	-0.042	0.876	0.691	0.003
IFN-γ	0.115	0.660	-0.152	0.559	-0.118	0.653	0.201	0.438
TNF-α	0.162	0.534	-0.628	0.008	-0.177	0.497	0.677	0.004
TNF-RI	-0.004	0.989	-0.451	0.071	0.054	0.839	0.605	0.012
TNF-RII	-0.009	0.972	0.311	0.224	0.167	0.521	0.422	0.093
CRP	0.108	0.680	-0.154	0.553	0.257	0.317	0.503	0.042
MCP-1	-0.049	0.854	-0.216	0.404	-0.156	0.551	0.701	0.002
SAA	0.294	0.252	0.230	0.372	0.512	0.038	-0.164	0.528
**Endothelial Markers**								
ICAM-1	0.213	0.410	-0.214	0.410	0.012	0.966	0.632	0.008
MMP-9	-0.043	0.871	-0.260	0.313	-0.179	0.492	0.495	0.045
VCAM-1	-0.285	0.267	-0.387	0.125	-0.180	0.489	0.681	0.003

Abbreviations: WBNs: whole blood neutrophils; LDNs: low density neutrophils; SYK: spleen tyrosine kinase. All data in the table are expressed as Spearman's correlation coefficient. Two-tailed $P \leq 0.05$ are deemed significant (bolded values).

Supplemental Table 8. Demographics of patients split by high and low levels of LDNs

**ClinicalVariables**	**Low LDNs (*n* = 18)**	**High LDNs (*n* = 17)**	***P*-value**
**Demographics**			
Age (years)	67 (11)	62 (16)	0.4
Sex			0.4
Male, *n (%)*	10 (56)	7 (41)	
Female, *n (%)*	8 (44)	10 (59)	
Race			0.8
White, *n (%)*	11 (61)	8 (47)	
Black, *n (%)*	3 (17)	4 (24)	
Asian, *n (%)*	3 (17)	3 (18)	
Unknown, *n (%)*	1 (5.6)	2 (12)	
**Hematologic Characteristics**			
White Blood Count, 10^3/uL, *mean (SD)*	11 (4)	16 (10)	0.03
Hemoglobin, g/dL, *mean (SD)*	9.92 (2.29)	9.92 (1.76)	>0.9
Platelets, /uL, *mean (SD)*	188 (125)	168 (85)	0.8
Serum Creatinine, mg/dL, *mean (SD)*	1.76 (1.12)	1.42 (1.00)	0.2
**Sepsis Severity**			
ICU Status			0.3
Non-ICU, *n (%)*	7 (39)	3 (18)	
ICU, *n (%)*	11 (61)	14 (82)	
SOFA Score, *mean (SD)*	4.06 (2.48)	4.71 (3.50)	0.8
Sepsis Status (at any point)			0.03
Sepsis, *n (%)*	11 (61)	4 (24)	
Septic Shock, *n (%)*	7 (39)	13 (76)	
Vasopressor (at time of blood draw)			0.2
Yes, *n (%)*	7 (39)	10 (59)	
No, *n (%)*	11 (61)	7 (41)	
Mechanical Ventilation			0.02
Yes, *n (%)*	1 (6)	7 (41)	
No, *n (%)*	17 (94)	10 (59)	
Acute Kidney Injury			0.4
Yes, *n (%)*	12 (67)	9 (53)	
No, *n (%)*	6 (33)	8 (47)	
Mortality			0.6
Yes, *n (%)*	1 (6)	2 (12)	
No, *n (%)*	17 (94)	15 (88)	
Length of Stay (LOS), days, *mean (SD)*	6.9 (3.1)	12.4 (8.1)	0.02
LOS After Blood Draw, days, *mean (SD)*	4.4 (3.2)	10.0 (8.0)	0.02
**Infection Source**			
Site of Infection			0.5
Skin/soft tissue, *n (%)*	2 (11)	2 (12)	
Intra-abdominal, *n (%)*	6 (33)	4 (24)	
Pneumonia, *n (%)*	3 (17)	5 (29)	

Urinary Tract, *n (%)*	3 (17)	3 (18)	
Other, *n (%)*	1 (5.6)	3 (18)	
Unknown, *n (%)*	3 (17)	0 (0)	
Organism			0.001
Polymicrobial, *n (%)*	1 (5.6)	5 (29)	
Gram Positive, *n (%)*	1 (5.6)	5 (29)	
Gram Negative, *n (%)*	3 (17)	3 (18)	
Viral, *n (%)*	1 (5.6)	2 (12)	
Fungal, *n (%)*	0 (0)	1 (5.9)	
Unknown, *n (%)*	12 (67)	1 (5.9)	

Abbreviations: ICU: Intensive care unit; SOFA: Sequential organ failure score; SD: Standard deviation. Values are reported as means ± SD for continuous variables and as n (%) for categorical variables. Statistical significance was determined by Wilcoxon rank sum test, Pearson's Chi-squared test or Fisher's exact test. *P* ≤ 0.05 were deemed significant (bolded values).

Supplemental Table 9. Demographics of patients split by high and low expression of SYK in LDNs

Clinical Variables	Low SYK⁺LDNs (*n* = 18)	High SYK⁺LDNs (*n* = 17)	*P*-value
**Demographics**			
Age (years)	71 (10)	58 (14)	0.004
Sex			0.4
Male, *n (%)*	10 (56)	7 (41)	
Female, *n (%)*	8 (64)	10 (59)	
Race			0.03
White, *n (%)*	14 (78)	5 (29)	
Black, *n (%)*	1 (5.6)	6 (35)	
Asian, *n (%)*	2 (11)	4 (24)	
Unknown, *n (%)*	1 (5.6)	2 (12)	
**Hematologic Characteristics**			
White Blood Count, 10^3/uL, *mean (SD)*	10 (4)	17 (10)	0.01
Hemoglobin, g/dL, *mean (SD)*	9.98 (2.02)	9.86 (2.07)	0.8
Platelets, /uL, *mean (SD)*	176 (118)	180 (96)	0.8
Serum Creatinine, mg/dL, *mean (SD)*	1.78 (1.11)	1.40 (1.01)	0.10
**Sepsis Severity**			
ICU Status			0.3
Non-ICU, *n (%)*	7 (39)	3 (18)	
ICU, *n (%)*	11 (61)	14 (82)	
SOFA Score, *mean (SD)*	3.94 (2.53)	4.82 (3.43)	0.5
Sepsis Status (at any point)			0.12
Sepsis, *n (%)*	10 (56)	5 (29)	
Septic Shock, *n (%)*	8 (44)	12 (71)	
Vasopressor (at time of blood draw)			0.2
Yes, *n (%)*	7 (39)	10 (59)	
No, *n (%)*	11 (61)	7 (41)	
Mechanical Ventilation			0.001
Yes, *n (%)*	0 (0)	8 (47)	
No, *n (%)*	18 (100)	9 (53)	
Acute Kidney Injury			0.4
Yes, *n (%)*	12 (67)	9 (53)	
No, *n (%)*	6 (33)	8 (47)	
Mortality			0.6
Yes, *n (%)*	1 (6)	2 (12)	
No, *n (%)*	17 (94)	15 (88)	
Length of Stay (LOS), days, *mean (SD)*	7.9 (6.8)	11.4 (6.0)	0.02
LOS After Blood Draw, days, *mean (SD)*	5.3 (6.7)	9.1 (5.9)	0.009
**Infection Source**			
Site of Infection			0.04
Skin/soft tissue, *n (%)*	2 (11)	2 (12)	
Intra-abdominal, *n (%)*	6 (33)	4 (24)	

Pneumonia, *n (%)*	1 (5.6)	7 (41)	
Urinary Tract, *n (%)*	5 (28)	1 (5.9)	
Other, *n (%)*	1 (5.6)	3 (18)	
Unknown, *n (%)*	3 (17)	0 (0)	
Organism			0.08
Polymicrobial, *n (%)*	2 (11)	4 (24)	
Gram Positive, *n (%)*	2 (11)	4 (24)	
Gram Negative, *n (%)*	4 (22)	2 (12)	
Viral, *n (%)*	0 (0)	3 (18)	
Fungal, *n (%)*	0 (0)	1 (5.9)	
Unknown, *n (%)*	10 (56)	3 (18)	

Abbreviations: ICU: Intensive care unit; SOFA: Sequential organ failure score; SD: Standard deviation. Values are reported as means ± SD for continuous variables and as n (%) for categorical variables. Statistical significance was determined by Wilcoxon rank sum test, Pearson's Chi-squared test or Fisher's exact test. $P \leq 0.05$ were deemed significant (bolded values).

Supplemental Table 10. Antibody and reagent list for flow cytometry experiments

Antibodies	Fluorophore	Clone	Company	Location	Cat. Number
MPO eFluor450	BV421	MPO455-8E6	ThermoFisher Scientific	Eugene, OR	48-1299-42
CD14	BV510	M5E2	BioLegend	San Diego, CA	301842
CD10	BV605	HI10a	BioLegend	San Diego, CA	312222
CD45	BV650	HI30	BioLegend	San Diego, CA	304044
CD33	BV711	P67.6	BioLegend	San Diego, CA	366624
CD15	BV786	W6D3 (RUO)	BD Biosciences	Franklin Lakes, NJ	568288
CD11b	FITC	ICRF44	BioLegend	San Diego, CA	301330
SYK	PE	4D10.2	BioLegend	San Diego, CA	644304
CD62L	PE/Dazzle	DREG-56	BioLegend	San Diego, CA	304842
CD63	PE-Cy7	H5C6 (RUO)	BD Biosciences	Franklin Lakes, NJ	561982
CD56 (NCAM)	APC	5.1H11	BioLegend	San Diego, CA	362504
CD2	APC	RPA-2.10	BioLegend	San Diego, CA	300214
CD3	APC	OKT3	BioLegend	San Diego, CA	317318
SIGLEC8	APC	7C9	BioLegend	San Diego, CA	347106
CD19	APC	HIB19	BioLegend	San Diego, CA	302212
CD16	R718 (RUO)	3G8	BD Biosciences	Franklin Lakes, NJ	566969
HLA-DR	APC/Fire 750	L243	BioLegend	San Diego, CA	980412
**Additional Reagents**					
Fixable Far Red Dead Cell Stain Kit	---	---	Invitrogen Life Technologies	Eugene, OR	L34974
FcR Blocking	---	---	Miltenyi Biotec	Bergisch Gladbach, Germany	130-059-901
Brilliant Stain Buffer	---	---	BD Horizon	Franklin Lakes, NJ	566349
Cytofix/ Cytoperm Kit	---	---	BD Biosciences	Franklin Lakes, NJ	554714
